# Acknowledgment to Reviewers of *Nanomaterials* in 2020

**DOI:** 10.3390/nano11020308

**Published:** 2021-01-25

**Authors:** Nanomaterials Editorial Office

**Affiliations:** MDPI AG, St. Alban-Anlage 66, 4052 Basel, Switzerland

Peer review is the driving force of journal development, and reviewers are gatekeepers who ensure that *Nanomaterials* maintains its standards for the high quality of its published papers. Thanks to the cooperation of our reviewers, in 2020, the median time to first decision was 14 days and the median time to publication was 33 days. The editors would like to express their sincere gratitude to the following reviewers for their precious time and dedication, regardless of whether the papers were finally published:
Abad, BegoñaLin, Hung-YinAbatzoglou, NicolasLin, JiahongAbbas, QamarLin, WeifengAbbasi, Qammer HussainLin, Yan-GuAbbrent, SabinaLin, Yang-weiAbdal-hay, AbdallaLin, Yi-LiAbdelkader, Amor M.Lin, ZhiweiAbdel-Mohsen, Abdellatif M.Linda, CattinAbdollahnejad, ZahraLinder, MarcAbdulhalim, IbrahimLindquist, NathanAbe, YoshioLindsay, Kenneth A.Abella, Pura AlfonsoLindström, TomAbellan, GonzaloLinkous, Clovis A.Aberoumand, SadeghLinkov, IgorAbidi, NoureddineLinzon, YoavAbiev, RufatLionetto, FrancescaAbrosimova, Galina YeLiopo, AntonAbu-Reziq, RaedLipatov, AlexeyAccardo, GraziaLipscomb, GlennAcik, Ilona OjaLiras, MartaAdam, VojtechLis, Jose Vicente RosAdamcakova-Dodd, AndreaLisco, FabianaAddepalli, BalasubrahmanyamLisi, NicolaAddou, RafikLisinska-Czekaj, AgataAdegoke, OluwasesanLisowska-Oleksiak, AnnaAdhikari, RajanLita, Adriana E.Adnan, MemicLitvin, Aleksandr P.Adrover, AlessandraLiu, Bo-TauAgar, ErtanLiu, Chao-LinAgarwal, MangilalLiu, Chee-WeeAgarwal, SumitLiu, Cheng-LiangAgeitos, Jose ManuelLiu, Cheng-YangAghazada, SadigLiu, Chen-WuingAgnoli, StefanoLiu, Chih-YiAgodi, AntonellaLiu, ChongyangAgostino, RaffaeleLiu, Chung-ChiunAgustin, DominiqueLiu, FeiAgustin, Molina OntoriaLiu, GuichengAgustina, AsenjoLiu, HaoAgut, MontserratLiu, JinmingAhlswede, ErikLiu, KaiAhmadivand, ArashLiu, KaimiaoAhmed, Mohammad BoshirLiu, MaoAhmmad, BashirLiu, Shyh-JiunAhn, DoyeolLiu, TianyuAhn, SanghyunLiu, Ting-YuAiello, Maria AntoniettaLiu, XiangshengAifantis, Elias C.Liu, XianjieAikawa, ShinyaLiu, XifengAimi, JunkoLiu, XinyuAiroudj, AissamLiu, YangAissa, BrahimLiu, YinAkbari Garakani, MohammadLiu, YingtaoAkhavan, OmidLiu, YunAkhlaghi, ShahinLizundia, ErlantzAkiba, IsamuLlosa Tanco, Margot A.Akimov, AndreyLloveras, PolAkimov, Sergey A.Lloveras, VegaAkitsu, TakashiroLo Presti, LeonardoAkiyama, ToruLo, Kuang YaoAkopova, TatianaLobato, KillianAksenov, SergeyLobo, CharleneAl-Anssari, SarmadLocatelli, AndreaAlapati, SureshLofaj, FrantišekAlapi, TundeLogothetidis, StergiosAlavi, SamanLonghi, MariangelaAlba, Maria D.Longo, AngelaAlbericio, FernandoLongo, Dario LivioAlbero, JosepLongo, SandroAlberto, VertovaLoontjens, Ton J. A.Albini, AngeloLopez Anton, RicardoAlcudia, AnaLópez, David MarreroAldakov, DmitryLopez, Felix A.Alegria, Elisabete C.B.A.Lopez, FrancescoAlessandro, CecconelloLopez, Gabriel AlejandroAlessandro, MattoniLopez, GartzenAlexiadis, AlessioLopez, ReneAlexiou, ChristophLopez-Cara, Luisa CarlotaAlfinito, EleonoraLópez-Cela, Juan JoséAli-Boucetta, HaneneLópez-Cornejo, PilarAllen, AndrewLópez-Garzón, Francisco J.Allen, Norman S.Lopez-Jornet, PiaAlluri, Nagamalleswara RaoLopez-Linares, FranciscoAl-Mamun, MohammadLópez-Lorente, Ángela I.Almásy, LászlóLópez-Ortega, AlbertoAlmeida, Bernardo GonçalvesLopez-Ramon, Maria VictoriaAlonso-Calvo, RaúlLópez-Rubio, AmparoAltmann, FriedrichLorè, Nicola IvanAlvarez, GonzaloLorenz, MichaelÁlvarez, MarLosada-Perez, PatriciaAlvarez, Nicolas JavierLosurdo, MariaAlvarez, Noe T.Lotnyk, AndriyÁlvarez-Alonso, PabloLott, DieterÁlvarez-Rodríguez, JesúsLour, Wen-ShiungÁlvaro, CaballeroLourenço, JoãoAlyokhin, AndreiLove, JohnAmabili, MarcoLovell, JonathanAmama, Placidus B.Low, NicholasAmani, MahmoodLozano, JesusAmara, HakimLu, Kuo-ChangAmat, EsteveLu, LiAmbrogi, VeronicaLu, MinghuaAmeen, SadiaLu, QinAmendola, EugenioLub, JohanAmendola, VincenzoLucarelli, CarloAmiens, CatherineLucchetta, GiovanniAmin, Mohamad HassanLucia, UmbertoAmine, AzizLuciani, GiuseppinaAmini, ShahrouzLuciano, GiorgioAmler, EvzenLuciano, RaimondoAn, HyosungLučić, BonoAn, Sang-MinLucklum, RalfAnasori, BabakŁuczak, JustynaAnastasescu, MihaiLue, Shing-Jiang JessieAnceschi, AnastasiaLuigia, SabbatiniAndablo-Reyes, Efrén AlbertoLuin, StefanoAndersen, Christian P.Luisa Grilli, MariaAndersson, Henrik A.Lukáč, PavelAndò, BrunoLukoyanov, AlexeyAndo, YoshitoLukyanov, Pavel A.Andón, Fernando TorresLuliński, PiotrAndreoli, CristinaLuliński, SergiuszAndreopoulou, Aikaterini K.Luna, ManuelAndreou, ChrysafisLundberg, HelenaAndreozzi, AssuntaLunelli, LorenzoAndrés González, Juan PedroLunov, OlegAndres, JuanLuo, JianAndronic, LuminitaLuo, LiuAng, Yee SinLuongo, GiuseppeAngela Barba, AnnaLuque-García, José LuisAngelakeris, MakisLützenkirchen-Hecht, DirkAngelici, Robert J.Luxbacher, ThomasAngelico, RuggeroLvova, LarisaAngelini, AngeloLy, Thuc HueAngelo, AngeliniLyalin, AndreyAngelov, BorislavLyons, John L.Angelova, AngelinaLyu, HailongAnglani, FrancaMa, Chih-MingAnicǎi, LianaMa, Yuan-RonAnisoara Peptu, CatalinaMa, ZhenAnitas, Eugen MirceaMaas, MichaelAnni, MarcoMacak, JanAnsón Casaos, AlejandroMaccaferri, NicolòAntal, IstvánMaccato, ChiaraAnthony, EdwardMacdonald, ThomasAnthony, RebeccaMachala, LiborAntoine, RodolpheMachrafi, HatimAntolini, ErmeteMacias, Rocio I RodriguezAnton, DavydokMaciejewska, MagdalenaAntoniac, IulianMacoas, ErmelindaAntonini, CarloMacucci, MassimoAntonino, FamulariMacutkevic, JanAntonioli, DiegoMączka, MirosławAntonov, MaksimMadhyastha, HarishkumarAntonova, Irina V.Madison, Alexey E.Anttu, NicklasMadsen, Steen J.Antunes, Joana C.Maeng, Han-JooApaydin, DogukanMaffeis, ThierryApetrei, ConstantinMagalhães, RicardoApostolova, TzvetaMagasinski, Alexandre N.Apostoluk, Aleksandra (Alexandra)Maggioni, DanielaAppell, MichaelMagklara, AngelikiAprili, MarcoMagna, GabrieleAradilla, DavidMagni, MirkoArakawa, TaroMagni, PaoloArami-Niya, ArashMagnuson, MartinArao, YoshihikoMagyari, KlaraAraya, Víctor CarrielMahdi, SammyArbelaiz, AitorMahian, OmidArciello, AngelaMahmood, AsifArčon, IztokMahmood, NasirArdekani, Mohammad MoghimiMahon, Peter J.Ariga, KatsuhikoMahony, Timothy JohnArinstein, ArkadiiMahy, Julien G.Arkadiusz, CiesielskiMailis, SakellarisArkas, MichaelMaimistov, Andrei I.Armbruster, UdoMaio, AndreaArmentano, IlariaMaiolo, LucaArnaut, Luís GuilhermeMaisetta, GiuseppantonioArnold, Donna C.Majewski, Jacek A.Arnusch, Christopher J.Majewski, LeszekArrabito, Giuseppe DomenicoMajewski, PawelArrais, AldoMakaremi, MeysamArrigo, RossellaMakarevich, PavelArruda, Thomas M.Makaryan, TaronArshad, AdeelMaki, YasuyukiArtemev, AndreiMakvandi, PooyanArtiaga, Ramón PedroMalagon, FranciscoArul, Narayanasamy SabariMalakhov, DmitriAsadi, AminMalandrino, GraziellaAsadnia, MohsenMalanga, MiloAsayama, ShoichiroMalankowska, AnnaAsensio, Maria-CarmenMalashicheva, AnnaAshrafi, AmirmansoorMalik, AdityaAshuri, MaziarMalik, RituAsmatulu, RamazanMalik, SharaliAšmontas, SteponasMalikan, MohammadAssadi, AymenMalka, DrorAssaf, Khaleel IbrahimMalkin, AlexanderAssmann, MarcMallamace, DomenicoAsuri, PrashanthMalusà, EligioAtabaev, Timur Sh.Mamajanov, IrenaAtanase, LeonardMamat, ConstantinAtchudan, RajiMamiya, HiroakiAtienzar, PedroMammeri, FaynaAtkinson, IrinaMan, AdrianAtuchin, VictorManakhov, AntonAubry, SylvieManakov, AndreyAvellan, AstridManariotis, Ioannis D.Aversa, LucreziaManconi, MariaAvgouropoulos, GeorgeManeiro, MarcelinoAvino, PasqualeManera, Maria GraziaAvnir, DavidManeuski, DimaAwad, Mohamed M.Mangiapia, GaetanoAwni, Rasha A.Mani, Ganesh KumarAwual, Md. RabiulMani, GovindasamyAyers, PaulManière, CharlesAymonier, CyrilManjunatha Reddy, Gollapalli NarayanaAyukawa, YasunoriMankin, Richard W.Ayyavoo, JayalakshmiMannino, SaverioAzarnia Tehran, DomenicoMANNO, Daniela ErminiaAzevedo, Nuno F.Mansfeld, DmitryAznar, ElenaManshina, AlinaAzzawi, MayMansoori, AliBabarao, RavichandarMantione, DanieleBabeva, TsvetankaMao, HanbinBabilas, RafałMao, YuanbingBabincová, MelániaMaqbool, MuhammadBabu, AnishMarabelli, FrancoBabu, BathulaMaragò, Onofrio M.Bacakova, LucieMarais, StephaneBaccarani, GiorgioMarakatti, VijaykumarBacciocchi, MicheleMaranghi, FrancescaBach, HoracioMarchetti, BarbaraBachman, John ChristopherMarchiol, LucaBadawy, Amro ElMarchionni, AndreaBaddorf, Arthur P.Marchuk, OleksandrBadea, MihaelaMarcì, GiuseppeBadilescu, SimonaMarciniak, ŁukaszBae, DowonMarconcini, PaoloBae, Ki HyunMarcos, MarcosBae, Tae-HyunMarcus, Matthew A.Baek, YoungbinMargaritis, LukasBaena, Francisco ManuelMǎrginean, GabrielaBaev, AlexanderMaria Grazia, SarpietroBaeza, MireiaMarianecci, CarlottaBago, Juli R.Mariani, StefanoBagryanskaya, ElenaMarić, MilanBahat-Treidel, EldadMarichy, CatherineBai, JinboMariggiò, Maria A.Bai, ShuhuaMarin, CatalinBaia, MonicaMarin, EliaBaibarac, MihaelaMarín, Josefa IsasiBaida, FadiMarin, LuminitaBaingane, AnkitMarín, María LuisaBaino, FrancescoMarin, Ricardo MallaviaBajda, TomaszMarinas, AlbertoBaji, AvinashMarinoiu, AdrianaBakandritsos, AristeidisMarinova, KrastankaBaker, PeterMarinova, VeraBakkers, ErikMarinucci, LorellaBakonyi, ImreMárk, Géza IstvánBala, CameliaMarkoutsa, EleniBalakina, MarinaMarkowitz, JosephBalalaeva, Irina V.Markowska-Szczupak, AgataBalandin, Alexander A.Marmiroli, BenedettaBalasingam, Suresh KannanMarmiroli, MartaBalasoiu, MariaMarmiroli, NelsonBalázsi, CsabaMarotti De Sciarra, FrancescoBalcar, HynekMarović, DanijelaBald, IlkoMarquardt, DrewBaldermann, AndreMarques, CarlosBaldino, LuciaMarques, PaulaBaldomir, DanielMárquez, FranciscoBaldoví, José J.Marradi, MarcoBalen, BiljanaMarrero-López, DavidBalestrazzi, AlmaMarszalek, MartaBalis, NikolaosMartel, BernardBallesta-Claver, JulioMartella, ChristianBallesté, Ruben MasMartin Galan, AidaBalme, SébastienMartin Solans, SantiagoBalzer, FrankMartín, CristinaBan, IrenaMartin, Jose MiguelBanach, MarcinMartinelli, AndreaBanchelli, MartinaMartinelli, EnzoBandala, Erick R.Martínez Prieto, Luis MiguelBandodkar, Amay J.Martínez Rodrigo, JavierBandyopadhyay, SmarakMartinez, LidiaBanerjee, WritamMartinez-Calvo, MiguelBanfi, FrancescoMartínez-Guitarte, José-luisBanks, Craig E.Martinez-Martinez, DiegoBanti, Christina N.Martínez-Martínez, VirginiaBao, AndeMartínez-Pedrero, FernandoBao, YinyinMartínez-Prieto, Luis M.Baptista, Pedro VianaMartínez-Tong, Daniel E.Barata, Joana F.B.Martin-Garcia, BeatrizBarbarin, YohanMartin-Gullón, IgnacioBarbato, Felicia Carla TizianaMartino, MarcoBarbeck, MikeMartino, SabataBarbera, JoaquinMartín-Ramos, PabloBarbetta, AndreaMartín-Rapún, RafaelBarbier, AntoineMartins, AlbinoBarbillon, GrégoryMartins, José A.Barczak, MariuszMartins, LuisaBardi, GiuseppeMartins, RodrigoBarea, Eva M.Martins, Rui C.Barek, JiriMartins, Verónica C.Barge, AlessandroMartín-Sánchez, JavierBarile, LucioMartucciello, NadiaBarille, RegisMartyński, TomaszBarison, SimonaMasaharu, NakayamaBarkoula, Nektaria MarianthiMasayuki, HagiwaraBaron, MarcoMasi, SofiaBarranco, AngelMasino, MatteoBarreca, DavideMassari, FrancescoBarreca, SalvatoreMassaro, MarinaBarriada, José LuisMastali, MohammadBarry, CarolMastrangeli, MassimoBar-Shir, AmnonMasubuchi, YujiBartkowiak, ArturMasuda, YoshitakeBartolomeo, Antonio DiMasumoto, HidetoshiBarucca, GianniMasuo, SadahiroBaryshnikov, Gleb V.Matei, CristianBasaran, CemalMatějka, VlastimilBasith, Iftekhar IbneMateo, ReyesBaskoutas, SotiriosMaterer, Nicholas F.Basoli, FrancescoMatis, KostasBastida, AgathaMativenga, MalloryBastús, NeusMato, SoniaBatmunkh, MunkhbayarMatos, InêsBator, GrażynaMatras-Postolek, KatarzynaBattaglia, VincentMatsuda, TomokiBattiato, AlfioMatsuhisa, NaojiBauer, Elvira M.Matsui, JunBäumler, HansMatsuo, TakashiBausells, JoanMatsuura, KazunoriBayraktar, EminMatteis, Fabio DeBazylinska, UrszulaMatthews, LorinBazzan, MarcoMatthews, PeterBeall, GaryMattsson, Mats-OlofBear, Joseph C.Matwijczuk, ArkadiuszBechelany, MikhaelMauk, Michael G.Beck, Hans ChristianMauriz, ElbaBecucci, MaurizioMaurizi, LionelBednarz, SzczepanMautner, AndreasBedon, ChiaraMaver, TinaBeebe, StephenMavromoustakos, ThomasBégin, DominiqueMaye, Mathew M.Begin, SylvieMayer, ChristianBeharry, Kay D.Mayo, KevinBehnamian, YasharMazurkiewicz-Pawlicka, MartaBeiras, RicardoMazzaglia, AntoninoBelardini, AlessandroMbata, GeorgeBelessi, VassilikiMcAfee, MarionBella, FedericoMcDonald, ArmandoBellani, SebastianoMcGettrick, JamesBellardita, MariannaMcMahon, Jeffrey M.Bellisario, DeniseMcMillin, Kenneth W.Belloncle, ChristopheMcPeak, Kevin M.Bellos, EvangelosMęczyńska-Wielgosz, SylwiaBellucci, AlessandroMedforth, Craig J.Bellucci, StefanoMedici, SerenellaBelonoshko, AnatolyMedvecky, LubomirBenčan, Andreja GolobMeen, Teen-HangBending, SimonMehrali, MehdiBenedetti, AlessioMehrpouya, MehrshadBenedetti, ElisabettaMei, BastianBenedikovic, DanielMeijide, FranciscoBenetti, DanieleMelchionna, MicheleBenim, Ali CemalMele, GiuseppeBenoit, DenizotMelguizo-Guijarro, ManuelBenseman, TimothyMelin, FredericBerdonosov, PeterMélinon, PatriceBerenjian, AydinMelnik, RoderickBerent, KatarzynaMelone, Mariarosa Anna BeatriceBergamaschi, EnricoMeloni, EugenioBerger, Daniela CristinaMelrose, JamesBerkó, AndrásMendez, Ana MariaBermudez, María DoloresMenendez, MiguelBerna, JoseMeng, ChaoBernardi, EttoreMeng, TobiasBernardini, SandrineMeng, XiangchaoBernardos, AndreaMenger, Fredric M.Bernards, MatthewMenichetti, LucaBernascon, LeonardoMenichetti, StefanoBernik, SlavkoMenini, MariaBernstein, ElliotMenon, JyothiBert, NikolayMenzel, StephanBertel, ErminaldMenziani, Maria CristinaBerto, FilippoMeo, Paolo LoBertolotti, MarioMera, GabrielaBerton, PaulaMerabia, SamyBertoncello, PaoloMerah, OthmaneBertrand, BenoîtMercone, SilvanaBertsch, PaulMere, ArvoBessais, LotfiMereshchenko, Andrey S.Bessergenev, Valentin G.Merlino, AntonelloBessmertnykh-Lemeune, AllaMeroni, DanielaBesteiro, Lucas VazquezMerrin, JackBettache, NadirMesaros, AmaliaBettazzi, FrancescaMeškys, RolandasBettini, SimonaMessina, FabrizioBettotti, PaoloMessina, RiccardoBezdetnaya-Bolotine, LinaMessori, LuigiBhalla, NikhilMestres, NarcísBharti, NeelamMetha, Gregory F.Bhatt, PriyankaMeyer, Daniel J.M.Bhattacharya, PratipMeyer, FlorentBhaumik, AnaghMeziani, MohammedBhowmik, Pradip K.Meziani, YahyaBianchi, AntonioMezzi, AlessioBianchi, MassimilianoMia, MozammelBianconi, FrancescoMicha, DimitraBidulská, JanaMichail, ChristosBieliński, Dariusz M.Michel, Carlos R.Biernat, JanMicheletti, ChristianBiffi, Carlo AlbertoMichiels, CarineBigall, NadjaMichl, ThomasBigoni, DavideMichna, AnetaBikiaris, DimitriosMiculescu, FlorinBilewicz, AleksanderMičušík, MatejBilia, Anna RitaMidya, RivuBini, FabianoMiglietta, Maria LuciaBini, MarcellaMihailescu, Ion N.Birch, BrianMihály, JudithBirke, Ronald L.Mihaly, MariaBirowosuto, Muhammad DanangMikhail, KirillinBisio, FrancescoMikhailov, Oleg V.Bisker, GiliMikolajczyk, AlicjaBiswas, ChandanMilanesio, MarcoBjorklund, RobertMilani, PaoloBlach, Jean-FrançoisMilano, FrancescoBłachowicz, TomaszMilasius, RimvydasBlagbrough, Ian S.Milavetz, BarryBlaikie, RichardMilcarek, ChristineBlanco, AngelesMile, IvandaBlanco-Valera, Maria TeresaMilke, RalfBlasco, JulianMiller, ReinhardBłasiak, SławomirMills, DavidBlaszczuk, ArturMilowska, KarolinaBlau, WernerMin, XinBlayo, AnneMinakshi, ManickamBlinova, IrinaMinami, IchiroBloch, WitoldMinamikawa, TakeoBloh, JonathanMinamiki, TsukuruBloise, AndreaMinarchick, ValerieBlokhin, Alexander M.Minas, GraçaBlundell, StephenMincheva, RosicaBoanini, ElisaMindaugas, GedvilasBoarino, LucaMindemark, JonasBobadilla, Luis F.Minea, Alina AdrianaBober, PatrycjaMínguez Vega, GladysBobet, Jean LouisMinin, Igor V.Boca, SandaMinko, SergiyBocchetta, PatriziaMintcheva, NeliBochenek, DariuszMinucci, SergioBochenkov, Vladimir E.Miola, MartaBodaghi, MahdiMiotello, AntonioBoddapati, LoukyaMiquelard-Garnier, GuillaumeBodo, EnricoMIRABELLA, SalvoBoeffel, ChristineMiranda, João MárioBoffi, AlbertoMirey, GladysBogani, LapoMirkhani, Seyyed AlirezaBogdanovskaya, VeraMiroshnichenko, AndreyBoggaram, VijayMiscuglio, MarioBoggio, ElenaMishra, NeerajBoghosian, SoghomonMishra, Pawan KumarBognár, GabriellaMishra, Sandeep KumarBohinc, KlemenMishra, ShashankBohm, SivasambuMishra, Yogendra KumarBoichot, RapahelMisiakos, KonstantinosBoix, Pablo P.Missell, FrankBoldt, RegineMistrik, JanBolivar, JuanMisture, Scott T.Bollella, PaoloMitchell, AndrewBolton, JamesMitchell, DrakeBon, VolodymyrMitchell, Scott G.Boncel, SlawomirMitomo, HideyukiBondarenko, OlesjaMitoraj, MariuszBondareva, Nadezhda S.Mitropoulos, Athanasios C.Bonduelle, ColinMittova, Irina YaBonelli, BarbaraMiyafuji, HisashiBonetta, SaraMiyauchi, MasahiroBonferoni, Maria CristinaMiyazaki, ToshikiBoni, Maria RosariaMiyoshi, DaisukeBonifacio, AloisMizoshita, NorihiroBonnet, Célia S.Mizsei, JanosBonomo, MatteoMo, YiqunBonyár, AttilaMoad, GraemeBoraschi, DianaMochizuki, ShinichiBorfecchia, ElisaModica, MariaBorges, João PauloMoein, Mohammad MahdiBorhan, Adrian IulianMogielnicki, AndrzejBorkow, GadiMohajernia, ShivaBorm, PaulMohammed, AltafBorodi, GheorgheMohammed, Hussein A.Boronin, Andrei I.Mohammed, YousufBorriello, AnnaMohanram, HariniBörrnert, FelixMohles, VolkerBortolotti, Carlo A.Moineau-Chane-Ching, Kathleen I.Borys, KrzysztofMoiseev, SergeyBorysiewicz, Michał AdamMoissette, AlainBosakova, ZuzanaMokhov, Evgeniy N.Boscá, FranciscoMolas, MaciejBoschetto, FrancescoMoldovan, AntoniuBoshir Ahmed, MohammadMolina, Carmen BelénBoshkov, NikolaiMolíns-Legua, C.Botiz, IoanMoloney, Mark G.Boukha, ZouhairMomekova, Denitsa B.Boukos, Nikos K.Momeni, KasraBoulesbaa, AbdelazizMonai, MatteoBouquillon, SandrineMonavarian, MortezaBourel-Bonnet, LineMondal, BikashBourillot, EricMondal, KunalBouropoulos, NikolaosMondal, SudipBourson, PatriceMondeshki, MihailBoutinguiza, MohamedMonguzzi, AngeloBouvet, MarcelMonné, MagnusBouwer, James C.Monobe, HirosatoBouzouraa, Montassar BillehMonopoli, MarcoBoyer, DamienMontagne, AxelBoyer, SeverineMontalenti, FrancescoBozio, RenatoMontanari, RobertoBrack, AndréMonteil-Rivera, FannyBraconi, DanielaMonteiro, Jorge H.Bradac, CarloMonteiro, OlindaBraeuninger, PhilippMontes Morán, Miguel A.Braic, MarianaMontesarchio, DanielaBrancolini, GiorgiaMontes-García, VerónicaBranda, FrancescoMonti, AlessioBrandão, PaulaMonti, DanielaBrandbyge, MadsMonti, DonatoBrandi, FernandoMonti, MarcoBratina, GvidoMonticelli, OriettaBravin, AlbertoMoon, Dae WoonBredikhin, VladimirMoon, Geon DaeBredow, ThomasMorak-Młodawska, BeataBrehm, MartinMorales, EnriqueBreidenstein, BerndMorales-Torres, SergioBreitung, BenMoralli, DanielaBreslin, Carmel BMorasso, CarloBretcanu, OanaMorávková, ZuzanaBreton, LionelMorbidelli, LuciaBrettmann, BlairMordini, AlessandroBriedis, VitalisMoreau, JulienBrivio, FedericoMoreira, André FerreiraBrkic, DejanMoreira, Maria TeresaBrogi, SimoneMoreira-Teixeira, LilianaBroniatowski, MarcinMorena, FrancescoBrougham, DermotMoreno, InésBrouzgou, ΑngelikiMoret, FrancescaBruce, AliceMoretto, AlessandroBrückl, HubertMorganti, PierfrancescoBruno, ElenaMorgera, Alessandro FraleoniBrutti, SergioMori, WasukeBu, LintaoMorimoto, NobuyukiBubnov, AlexeyMorimoto, YasuoBuchet, ReneMoritz, MichałBuckner, Steven W.Morkoc, HadisBuckwell, MarkMoros, MaríaBudai-Szűcs, MáriaMorra, MarcoBueno, MoisesMortazavi, BohayraBuergers, RalfMortimer, MonikaBugaev, AramMosavi, AmirBuha, JokaMościcki, TomaszBuhot, ArnaudMoshizi, Sajad AbolpourBuijnsters, IvanMoshnyaga, VasilyBujak, PiotrMoskvin, Alexander S.Bujanovic, BiljanaMostafavi, MehranBulgariu, LauraMoszyński, DariuszBullon, PedroMotoyama, KeiichiBulusheva, LyubovMott, Derrick M.Bungau, SimonaMoultos, OthonasBunge, Christian-AlexanderMourdikoudis, StefanosBunker, Christopher E.Mourtas, SpyridonBunoiu, Octavian MădălinMouzakis, Dionysios E.Buonocore, FrancescoMoya, Antonio A.Buravkova, LudmilaMozdziak, PaulBureš, RadovanMrazek, JanBurlacu, AlexandrinaMrlík, MiroslavBurns, Jorge S.Mrówczyński, RadosławBury, WojciechMróz, WaldemarBusby, YanMu, QingxinBuschmann, Matthias H.Muench, FalkBusquets, Maria AntòniaMühlberger, MichaelBussolati, OvidioMukherjee, SoumyaButi, JacopoMukherjee, SudipButkute, RenataMukhopadhyay, TanmoyButt, Muhammad AliMüller, DannyByrne, HughMüller, GerhardBystrov, VladimirMULLER, RobertCabaj, JoannaMüller, Thomas ErnstCabaleiro, DavidMuniz-Miranda, FrancescoCabanelas Valcárcel, Juan CarlosMuniz-Miranda, MaurizioCabot, AndreuMuñoz, FranciscoCabot, Pere L.Munoz-Pinto, DanyCabral, Benedito J.C.Munteanu, BogdanCabrele, ChiaraMunteanu, Florentina-DanielaCabrera, HumbertoMünzenrieder, NikoCacciotti, IlariaMuramatsu, AtsushiCada, MartinMuramatsu, HiroyukiCadar, OanaMurase, NorioCadatal-Raduban, MarilouMuratore, ChristopherCaddeo, CarlaMuravyev, Nikita V.Cadierno, VictorioMurcia Mesa, Julie JoseaneCadillon, LuisMurcia-López, SebastiánCaggiano, AntonioMurray, KeithCaggiati, AlbertoMusić, SvetozarCagli, CarloMusmarra, DinoCahay, Marc M.Musselman, KevinCai, ZhongyuMustafina, AsiyaCailliez, FabienMusumeci, ChiaraCairns, Elton J.Musumeci, TeresaCaiwei, ShenMuter, Olga A.Calabrese, LuigiMuzioł, Tadeusz MikolajCalabria, DonatoMuzza, MarinaCalamante, MassimoMyers, Timothy G.Calarco, AnnaMyśliwiec, JarosławCalatayud, David G.Na, Jun-HeeCaldas, PauloNa, KyungsuCalifano, ValeriaNabiałek, MarcinCalin, ManuelaNabil, BouaziziCalina, DanielaNacci, AngeloCalogero, AntonellaNada, Amr A.Calpena, Ana C.Nadolna, JoannaCalvaresi, MatteoNagai, MasayaCalvete, MarioNagai, NoriakiCalvo, FlorentNagamine, KuniakiCambier, SébastienNagane, SatyawanCamerlingo, CarloNagao, DaisukeCameron, DavidNagao, TadaakiCametti, CesareNagaoka, ShojiCaminati, GabriellaNagatani, NaokiCamino, GiovanniNaghdi, SamiraCammas-Marion, SandrineNagy, LászlóCampanella, LuigiNair, AshwinCampbell, ColinNair, Sandeep SudhakaranCampbell, KrisNakabayashi, TakakazuCampello, PaulaNakajima, KenCampos-Martínez, JoséNakano, MasahiroCancelas, Jose A.Nakashima, TakuyaCaneschi, AndreaNakhchi, Mahdi ErfanianCanet-Ferrer, JosepNalbach, PeterCanetta, ElisabettaNalimov, Anton GennadyevichCanfield, ScottNallathamby, Prakash D.Cannas, MarcoNam, Ki MinCannon, Carolyn L.Nam, SeunghoonCano Luna, ManuelNand, AshveenCantalini, CarloNanjunda, Shivananju BannurCantele, GiovanniNanver, Lis K.Cao, CuongNaraghi, MohammadCao, JianyunNarajczyk, MagdalenaCao, XudongNardecchia, StefaniaCapacchione, CarmineNardi, MonicaCapogni, MarcoNarita, FumioCapparè, PaoloNarojczyk, Jakub W.Cappellano, GiuseppeNasarre, PatrickCappelletti, GiuseppeNasillo, GiorgioCaprara, SergioNasiri, NoushinCapron, MickaelNassiopoulou, AndroulaCapsoni, DorettaNastase, FlorinCaputo, GregoryNatale, PaoloCarabineiro, SóniaNatali Sora, IsabellaCaramella, Carla MarcellaNaushad, MuCarata, ElisabettaNavarro, ElenaCarbajo, JaimeNavarro-Quezada, AndreaCarballal, Ramon NovoaNavid, KnejadCarbone, ClaudiaNaya, Shin-ichiCarda Castelló, Juan B.Nayak, Usha Y.Cardia, Maria CristinaNazarchuk, EvgenyCaridad, JoseNazemi, AliCarli, StefanoNeagu, AdrianCarlucci, ClaudiaNechifor, GheorgheCarmona, ManuelNedelec, Jean-MarieCarniato, FabioNehmé, ReineCaron, ArnaudNeisiany, Rasoul EsmaeelyCarosio, FedericoNekvindova, PavlaCarotenuto, GianfrancoNemes, Norbert M.Carraro, MauroNemnes, George AlexandruCarravetta, VincenzoNeophytou, NeophytosCarregal-Romero, SusanaNeri, GiovanniCarrete Montaña, JesúsNesterov, DmytroCarretero-Palacios, SolNetskina, OlgaCarta, MariolinoNeufeld, OferCaruntu, GabrielNeufurth, MeikCaruso, GerardoNeugebauer, DorotaCarvajal, Joan JosepNeverov, Vladimir N.Casado-Coterillo, ClaraNeves, ArmandoCasari, Carlo S.Neves, Cristina Sofia Dos SantosCasas Bedoya, AlvaroNeves, RuiCaschera, DanielaNevshupa, Roman ACasella, PatriziaNewton, Graham N.Caseri, WalterNeyts, KristiaanCasey, SeanNgamchuea, KamonwadCasnati, AlessandroNghia, Truong PhuocCassani, Maria CristinaNguyen, Duc ThanhCassidy, John F.Nguyen, Hanh ThuyCastaldi, GiuseppeNguyen, Khoi TanCastán, HelenaNguyen, Minh ThoCastanheira, Elisabete M.S.Nguyen, Nhat TruongCastellanos, SoniaNguyen, Nhung H.A.Castellanos-Gomez, AndresNguyen, TrieuCastellino, MicaelaNguyen, Van KhanhCastillo, OscarNiaura, GediminasCastro-Muñoz, RobertoNicholson, DavidCasula, MicheleNicholson, John W.Cataldo, AntoninoNiclós-Gutíerrez, JuanCataudella, VittorioNicoletta, Fiore PasqualeCatauro, MichelinaNiedzialkowski, PawelCatelani, TizianoNiedziółka-Jönsson, JoannaCatizzone, EnricoNiemczak, MichalCattoën, XavierNiemi, TapioCautela, DomenicoNieszporek, KrzysztofCauwe, MaartenNightingale, AdrianCavaliere, EmanueleNiidome, TakuroCavallaro, GiuseppeNikolakakis, IoannisCavani, FrancescoNikolaou, SymeonCavigli, LuciaNikolay V., SidorovCazzanelli, MassimoNikolelis, Dimitrios P.Cea Mingueza, PilarNikulov, Aleksey V.Cebecauer, MarekNilsen, OlaCeccarelli, GabrieleNima, Zeid A.Cecchet, FrancescaNischang, IvoCecilia, Juan AntonioNishikiori, HiromasaCefalas, Alciviadis-ConstantinosNishiwaki, HisashiCelano, UmbertoNisnevitch, MarinaCellesi, FrancescoNistor, Ileana DenisaČėnas, NarimantasNitschke, MirkoCenian, AdamNitulescu, MihaiCenteno, AnthonyNocca, GiuseppinaCepeda, JavierNoel, Jean-MarcCeresoli, DavideNoël, SébastienCernea, MarinNogales, EmilioČerný, MiroslavNoma, NaokiCerqueira, Miguel ÂngeloNomura, MikihiroCerrada, ElenaNomura, ShintaroCervelló, Gemma GutiérrezNoor, NuruzzamanČervinka, CtiradNoordermeer, Jacques W.M.Cesário, RuteNordblad, PerCha, ChaenyungNordin, JoelCha, Sang-HoNorek, MałgorzataChaboussant, GregoryNorkus, EugenijusChai, Kyu YunNorton, MichaelChaldyshev, VladimirNosoudi, NasimChammingkwan, PatchaneeNotargiacomo, AndreaChandra, SanjeevNoubactep, ChicgouaChandrasekaran, SwethaNouvel, CécileChang, Chia-ChenNovikov, AlexeyChang, Chien-ChungNovikov, Sergey M.Chang, Chih-WeiNovio, FernandoChang, Chi-JungNovotna, VladimiraChang, Ching-yaoNowak, AdrianaChang, Dong WookNowak-Sliwinska, PatrycjaChang, Jih-HsingNowicki, Margaret A.Chang, Sheng-PoNtalli, Nikoletta G.Chang, SheryNunes, Carla D.Chang, Ting C.Nunzi, FrancescaChang, Yu-ChengO’Rear, EdgarChang-Jian, Cai-WanObata, YasukoChantana, JakapanObraztsov, AlexanderChaput, FrédéricObraztsova, Elena D.Charalampidis, GeorgiosOchiai, BungoCharas, AnaOchieng, JosiahCharles Andrew, DowningOćwieja, MagdalenaCharpentier, LudovicOezkan, SeldaChassaing, StefanOgasawara, ToruChattopadhyay, SaurabhOgawa, ShinpeiChaudhary, VarunOgino, IsaoChaudhry, Mohammed QasimOh, Jae-MinChełkowska, GrażynaOh, JinwooChen, Chia-YunOh, JonghyunChen, Chuh-YungOh, JuwonChen, Chun-ChiOh, Won ChunChen, DaiqinOhashi, MasashiChen, DanielOhisa, SatoruChen, GuanyingOhlckers, PerChen, Hone-ZernOhmi, Shun-ichiroChen, Jhy-DerOhmura, TakahitoChen, Jian-LianOhno, SeigoChen, Kuan-RenOhtaka, AtsushiChen, Lung-ChienOhto, KeisukeChen, PeilinOikonomou, Evdokia K.Chen, QianOk, Jong G.Chen, QuarkOkajima, JunnosukeChen, Shih-YunOkamoto, MasamiChen, Tao-HsingOkazaki, JojiChen, WeiOkazaki, ToshiyaChen, Wen-ChengOkoshi, MasayukiChen, XiangzhongOku, TakeoChen, XiaoboOlaizola, Santiago M.Chen, XuechengOlejniczak, Agnieszka B.Chen, YijiaOlejniczak, IwonaChen, Yong-SongOlesiak-Banska, JoannaChen, Yuan-TsungOlga, GuselnikovaChen, Yu-ChangOlivares, JimenaChen, Yun-ShengOliveira, VítorCheng, Chih-ChiaOllila, SamuliCheng, Chung-WeiOlmos, DaniaCheng, Chun-HuOlyaee, SaeedCheng, Fong-YuOmastová, MáriaCheng, GuanghuaOms-Oliu, GemmaCheng, Hsin-MingOmura, YasuhisaCheng, Hsu-HuiOnaka, SusumuCheng, HuanyuOniga, IlioaraCheng, I. FrancisOno, AtsushiCheng, JipengOoi, Chin HongCheng, JipingOpitz, Alexander K.Cheng, Yang-TseOpitz, JörgCheng, Yuan-BinOpra, DenisCheng, ZhengdongOprea, AlexandruCherkaoui, KarimOprea, OvidiuChernysheva, Maria G.Orbach, RonChesnel, KarineOreshonkov, AleksandrChew, Weng ChoOrge, CarlaChiang, Cheng-TienOrlovskii, YuriiChiang, Chi-LingOrmeci, AlimChiang, Ming-HsiOrmellese, MarcoChiang, NaihaoOrozco, JahirChiang, Po-ChangOrtega, José MarcosChiasera, AlessandroOrtgies, Dirk H.Chieh, Jen-JieOrtí, EnriqueChien, Feng-TsoOrtore, Maria GraziaChien, Hsiu-WenOrudzhev, FaridChiesa, EnricaOsakada, YasukoChih Wei, LuoOschatz, MartinChikoidze, EkaterineOsella, SilvioChillura Martino, DeliaOsipov, AndreyChin, Alan H.Osipov, VladimirChinappi, MauroOsses, NelsonChiodoni, AngelicaÖsterholm, AnnaChiolerio, AlessandroOsvath, ZoltanChiper, ManuelaOtero, MartaChipouline, ArkadiOtomo, JunichiroChirolli, LucaOttolenghi, LiviaChirumamilla, ManoharOtyepka, MichalChiu, Chih-WeiOu, JianzhenChiu, Fang-ChyouOuerghi, AbdelkarimChiu, Nan-FuOuvrard, AimericChiu, Tai-chiaOuyang, ZiChizhik, Alexey I.Oyedele, AkinolaChmielarz, LucjanOzaydin, FatihChmielarz, PawełPacheco Tanaka, David AlfredoCho, Cheong-WeonPaciorek-Sadowska, JoannaCho, Eun JeongPaczesny, JanCho, Eun-BumPadamati, Ramesh BabuCho, In SunPadil, Vinod V.T.Cho, Jung SangPadmanabhan, VenkatCho, Man HoPadnya, PavelCho, Minhaeng HaingPaduszynska, MalgorzataCho, Sang WanPaganelli, SimoneCho, So-HyePagani, StefaniaCho, SooyeonPaganini, Maria CristinaCho, SunghunPagano, CinziaCho, SuyeonPagano, StefanoChocyk, DariuszPagliara, StefaniaChoi, Duk-YongPaik, TaejongChoi, Hyun ChulPaik, UngyuChoi, Jae HoonPaine, KevinChoi, Jane RuPaineau, ErwanChoi, Jeong-WooPak, ChanhoChoi, JonghoonPak, SangyeonChoi, JungilPakrashi, VikramChoi, JungwookPal, SudeshnaChoi, Myong YongPalamà, IlariaChoi, Seok KiPalanisamy, SelvakumarChoi, Sung-HwanPalau, AnnaChoi, Yong GyuPalazon, FranciscoChoi, Yoon-SeukPalermo, EdmundChoi, Young WhanPalinko, IstvanChong, Parskson Lee GauPall, EmokeChoo, JaebumPallavicini, PiersandroChorazy, SzymonPalmans, Anja R.A.Choudhury, SoumyadipPalmas, SimonettaChow, JamesPalmieri, ValentinaChrcanovic, BrunoPalomares, Antonio EduardoChrissopoulou, KiriakiPalomo, JoseChristensen, Jørn B.Paluch, Andrew S.Christensen, MogensPalyi, GyulaChristoforidis, KonstantinosPalzer, StefanChrobak, ArturPamies, RamonChrobok, AnnaPana, OvidiuChu, BenjaminPanchapakesan, BalajiChu, Chun HungPanczyk, TomaszChuang, Er-YuanPandey, RaviChul Wee, LeePané Vidal, SalvadorChung, DeborahPanek, PiotrChung, Jin SukPanek, RafalChung, Kung-MingPaneth, PiotrChung, Sheng-HengPang, YoonsooChwastek, KrzysztofPanich, Alexander M.Chyrvony, VladimirPanin, SergeyCiampi, SimonePankratov, AndreyCicala, GianlucaPanneerselvam, RajapandiyanCicchi, StefanoPanomsuwan, GasiditCicciù, MarcoPanos, PapagiannakopoulosCiccone, MarcoPant, BishweshwarCiccotosto, Giuseppe D.Pantano, PaulCichomski, MichalPanunzi, BarbaraCichowska-Kopczyńska, IwonaPanzarasa, GuidoCieśliński, JanuszPaolella, AndreaCifone, Maria GraziaPaolo, BollellaCiftja, OrionPaolone, AnnalisaCigler, PetrPap, JózsefCillero-Pastor, BertaPapaccio, GianpaoloCîmpean, AnişoaraPapaderakis, Athanasios A.Cinteza, Otilia LudmilaPapadimitriou, TheodotiCioffi, NicolaPapadogiannis, NektariosCiolaciu, DianaPapadopoulos, Antonios N.Cioncolini, AndreaPapagelis, KonstantinosCipak Gasparovic, AnaPapageorgiou, George Z.Cipak, LubosPapakonstantinou, Christos G.Ciríaco, LurdesPapanikolaou, NikolaosCirillo, GiuseppePapanikolaou, NikosCirlin, GeorgePapathanassiou, Anthony N.Cirtoaje, CristinaPapavasiliou, JoanCismaru, AlinaPapavassiliou, DimitriosClancy, AdamPaquette, BenoitClarizia, LauraParajuli, DurgaClarot, IgorParakhonskiy, BogdanClayton, John D.Parale, Vinayak G.Clegg, Jack K.Paraoanu, Gheorghe SorinClemens, OliverParenago, Oleg P.Clement, JoachimParigger, ChristianClement, YuenParis, JuanClift, MartinParisi, FilippoCobianu, CornelPark, Byung-WookCochis, AndreaPark, Chul-hoCockayne, EricPark, ChulhwanCoduri, MauroPark, Deok-YongCoelho, Luís CarlosPark, Dong HyukCoelhoso, IsabelPark, GyungsoonCohen Stuart, MartienPark, JaehoonCohen, BoikoPark, Jea-gunCohen, YachinPark, Jeong YoungCoisson, MarcoPark, JeyoungColace, LorenzoPark, JinhyukColacurci, NicolaPark, Jin-SooColla, Eugene V.Park, JuhyunCollini, ElisabettaPark, Jung-hyunCollins, ScottPark, Ki SooColon, JorgePark, Kwi-IlColumbu, StefanoPark, KyeongsoonComan, CristinaPark, Min BumComesaña, RafaelPark, Min HyukComes-Franchini, MauroPark, MiraCompagnone, DarioPark, Myoung-HwanCompton, RichardPark, NokukConcellon, AlbertoPark, Sang JoonConcli, FrancoPark, Sang-WonConnolly, James P.Park, Seong-JikConstantinescu, Catalin-DanielPark, Sung-KyuConte, ClaudiaPark, WooramConti, BicePark, Young WookConvertino, AnnalisaPark, Young-KwonCoppola, SaraParker, Stewart F.Corcione, Carola EspositoParkinson, PatrickCordani, MarcoParlińska-Wojtan, MagdalenaCoronado, Juan M.Parmeggiani, MatteoCorpino, RiccardoParra, RubenCorregidor, VictoriaParrilla Pons, MarcCorreia, ManuelaParrino, FrancescoCorrielli, GiacomoParsons, JasonCorrigan, Damion K.Parthenios, JohnCortés, EmilianoParvez, KhaledCortés, VicentePasán, JorgeCosco, DonatoPascual, LauraCosma, PinalysaPascuta, PetruCosta, Carlos MiguelPashirova, TatyanaCosta-Almeida, RaquelPashkin, OleksiyCostantini, AnielloPasini, DarioCostantino, FerdinandoPasinszki, TiborCosta-Rama, EstefaníaPaskaleva, AlbenaCoster, Peter DePaso, KristoferCotella, DiegoPasqua, LuigiCouce, María D.Passeri, DanieleCoudret, ChristophePassow, ThorstenCousins, Brian G.Pasta, SalvatoreCoutinho, PaulaPasternak, Taras P.Coutinho, Paulo José GomesPastero, LindaCovas, José AntónioPastore, CarloCox, DavidPastoriza-Santos, IsabelCoy, EmersonPaszkiewicz, SandraCozzoli, Pantaleo DavidePatanè, SalvatoreCragg, PeterPatel, KapilCranfield, CharlesPatel, RajanCreatore, MariadrianaPatel, RajkumarCrespi, AndreaPatel, SanjayCreton, StuartPatelli, AlessandroCretu, SpiridonPathak, RajeshCrezee, HansPathakoti, KavithaCrichton, WilsonPatolsky, FernandoCrisan, OvidiuPatra, Jaynta K.Crisp, Ryan W.Patroi, DeliaCristea, CeciliaPatroniak, ViolettaCristea, Mihaela DanaPatzelt, AlexaCristescu, RodicaPaul, RajibCristian, PetcuPaul, Suvash ChandraCroce, Anna CletaPaul, TitanCronin-Golomb, MarkPaul, UttamCrowley, PeterPauliukaite, RasaCrunteanu, AurelianPaulo, Pedro M.R.Crupi, VincenzaPaulsen, HaukeCruz, SandraPaulus, BeateCsapó, EditPaulusse, JosCsontos, MiklósPaun, Maria-AlexandraCtibor, PavelPaunesku, TatjanaCuberes, TeresaPavel, Octavian DumitruCucinotta, FabioPavelescu, Emil MihaiCucinotta, GiuseppePavesi, MauraCupellini, LorenzoPavlos, KlepetsanisĆurković, LidijaPawar, SachinCuscunà, MassimoPawashe, ChytraCuypers, DieterPawlak, AndrzejCzeppe, TomaszPawlak, MichałCzigany, ZsoltPawłat, JoannaCzyż, JarosławPeale, RobertD’Alfonso, LauraPeana, Massimiliano F.D’Amore, MarcelloPedersen, HenrikD’andrea, CristianoPedicini, RolandoD’Arienzo, MassimilianoPedraza, Luís CerdánD’Auria, FrancescoPedron, SaraD’Auria, MaurizioPedzich, ZbigniewD’Errico, GerardinoPedzinski, TomaszD’Orazio, AntonellaPeer, PetraD’Orazio, FrancoPeera, Shaik GouseDa Costa Ribeiro, Ana PaulaPeijnenburg, Willie J.G.M.Da Costa, Ana Maria Dos Santos RosaPeiner, ErwinDabrowski, JanuszPelin, MarcoDagmara, MalinaPeljo, PekkaDagvadorj, JargalsaikhanPellegrin, YannDai, NingPellegrino, FrancescoDale, SaraPellicer, EvaDalessandri, DomenicoPena, Maria MarjoretteDalhaimer, PaulPence, BrandtDamma, DevaiahPeng, Shu-FenDamron, Timothy A.Peng, ZhiliDams-Kozlowska, HannaPenkov, Oleksiy V.Dang, DongbinPenn, GregoryDaniel-da-Silva, Ana LuísaPepe, Giovanni PieroDaniele, SalvatorePeponi, LauraDaniele, StephanePeppel, TimDanko, MartinPerale, GiuseppeDantan, AurélienPerche, FedericoDantelle, GéraldinePerduca, MassimilianoDao, Van-DuongPereira, Ana LuisaDarafsheh, ArashPereira, LucianaDaripa, PrabirPereira, LuísDarling, SethPereiro, Ana B.Das, BhaskarPereiro, RosarioDas, GautamPerera, AureliénDas, NarottamPeres, NunoDas, ProdipPérez Colodrero, Rosario MercedesDas, SudipPerez Del Pino, AngelDash, BirajaPérez Tomás, AmadorDash, Saroj PrasadPérez, AntonioDassios, Konstantinos G.Pérez-Álvarez, LeyreDatta, ShubhashisPérez-Fernández, MaríaDaubon, ThomasPérez-Juste, IgnacioDavid, AdrianPérez-Luna, Victor H.David, Gabriela IuliaPérez-Prieto, JuliaDavid, Iulia GabrielaPerez-Sayans, MarioDavid, LuminitaPergher, SibeleDavidchack, Ruslan L.Periasamy, Arun PrakashDavis, FrankPérigo, Elio AlbertoDavis, Ryan D.Perioli, LuanaDe Almeida, Jose ManuelPerissutti, BeatriceDe Andrés, AliciaPeriyasamy, Arun PrakashDe Aza, Piedad N.Perlepes, SpyrosDe Bonis, AngelaPerlepes, Spyros P.De Caro, VivianaPerna, GiuseppeDe Castro, Carlos A. NietoPerov, NikolayDe Castro, SoniaPerova, Tatiana S.De Cesare, FabrizioPerrin, LaraDe Domenico, DarioPerrotti, VittoriaDe Dood, Michiel J.Perry, ChristopherDe Falco, GianluigiPersaud, KrishnaDe Freitas, Ana Cristina Cardoso Freitas LopesPerumal, SugunaDe Giglio, ElviraPescetelli, SaraDe Julián Ortiz, JesusPestryakov, AlexeyDe La Fuente, RaúlPetala, AthanasiaDe Lacalle, Luis N. LópezPeter, AncaDe Ligny, DominiquePeter, Van Der WalDe Luca, LuigiPetersen, ElijahDe Luca, PierantonioPetit, ChristopheDe Luna-Bertos, ElviraPetit, TristanDe Matteis, ValeriaPetráková, VladimíraDe Nalda, RebecaPetralito, StefaniaDe Pasquale, IlariaPetrič, MarkoDe Rosis, AlessandroPetrik, PeterDe Sio, LucianoPetrila, IulianDe Sousa, Célia TavaresPetrovski, ŽeljkoDe Stefano, LucaPetti, LuisaDe Tommasi, EdoardoPetukhov, Andrei V.Dea-Ayuela, María AuxiliadoraPetykiewicz, JanDeak, AndrasPeukert, DylanDebéda, HélènePeydayesh, MohammadDebnath, J.C.Pezeshki, AtiyeDecroly, AndréPezzella, AlessandroDeepagan, Veerasikku GopalPezzin, SergioDeflorian, FlavioPfeiffer, IlonaDeilmann, ThorstenPfeiffer, KristinDeimede, ValadoulaPhan, Hoang-PhuongDejneka, AlexandrPhan, Manh-HuongDel Buono, DanielePhilipose, UshaDel Gaudio, CostantinoPhilipsen, HaroldDel Real-Romero, Juan CarlosPhoenix, Stuart LeighDelfino, InesPiasecki, AdamDelgado, Ángel V.Piattelli, AdrianoDelgado-Aguilar, MarcPicca, Rosaria AnnaDelichatsios, Michael A.Piccirillo, ClaraDelimitis, AndreasPichat, PierreDell’Anna, Maria MichelaPicollo, FedericoDell’Olio, FrancescoPiconi, CorradoDella Pergola, RobertoPicos, RodrigoDella Ventura, BartolomeoPiedade, Ana PaulaDella Volpe, ClaudioPiekoszewski, WojciechDelogu, FrancescoPiel, GeraldineDelongchamp, DeanPielichowski, KrzysztofDelville, Marie-HelenePieraccini, StefanoDemchenko, AlexPierce, David M.Demetrescu, IoanaPiergiovanni, LucianoDemey, HaryPierson-Wickmann, Anne-CatherineDemirci, IbrahimPietrasik, JoannaDencheva, Nadya VasilevaPietropaolo, AdrianaDendrinou-Samara, CatherinePilan, LuisaDeng, WeiPilgrim, Wolf-ChristianDeniaud, AurélienPilot, RobertoDenkov, NikolaiPiña Capó, Mª NievesDennis, T. John S.Pina, SandraDerewiński, MirosławPinalli, RobertaDerrien, ThibaultPinčák, RichardDeschaume, OlivierPineda Rodríguez, TeresaDesgranges, LionelPineda, AntonioDesterke, ChristophePiñeiro, MartaDeville, SarahPinho, DianaDey, GangotriPinna, NicolaDhakar, Nilesh KumarPino, LidiaDhanjai, DhanjaiPinsino, AnnalisaDi Bartolomeo, AntonioPintauro, Peter N.Di Bella, ClaudiaPinto Da Silva, LuísDi Corato, RiccardoPinto, ArturDi Marco, AlessandroPinto, RicardoDi Maro, AntimoPiosik, JacekDi Martino, AntonioPiotrowska, Anna B.Di Martino, PieraPiotto, MassimoDi Nardo, FabioPiozzi, AntonellaDi Noto, VitoPiperno, AnnaDi Palma, LucaPiracha, AfaqDi Pasqua, Anthony J.Pisana, SimoneDi Stefano, AntonioPisaneschi, FedericaDiabaté, SilviaPisarek, MarcinDiana, RositaPisciotta, AlessandraDiaz Fernandez, YuriPiscitelli, FilomenaDíaz, EsperanzaPispas, StergiosDíaz, EvaPistone, AlessandroDiCarlo, David A.Piszczek, PiotrDichiara, AnthonyPiszczyk, ŁukaszDíez-Pascual, Ana MaríaPita, MarcosDijksman, J. FritsPitet, LouisDiliberto, SebastienPitilakis, AlexandrosDilley, Neil R.Piyasena, MenakeDimakis, EmmanouilPizzoferrato, RobertoDimakis, NicholasPlachetka, UlrichDimakopoulos, YannisPlamper, FelixDimesso, LucangeloPleşu, NicoletaDimic-Misic, KatarinaPlewa, JulianDimitratos, NikolaosPlonska-Brzezinska, MartaDimitri, RossanaPlotniece, AivaDimos, KonstantinosPoccia, NicolaDina, Nicoleta ElenaPochard, IsabelleDinca, ValentinaPodgornov, FedorDinescu, SorinaPodgornov, Fedor V.Ding, YapingPodgursky, VitaliDing, YongPodhorodecki, ArturDinh, ToanPodkościelna, BeataDini, LucianaPodraza, NikolasDinischiotu, AncaPoggi, GiovannaDiomede, FrancescaPoggini, LorenzoDippong, ThomasPogrebnjak, AlexanderDiring, StephanePogue, BrianDivitini, GiorgioPohl, PawelDjanashvili, KristinaPokutnyǐ, Sergey I.Djenizian, ThierryPolat, Emre OzanDo Rego, Ana Maria P.L.R. BotelhoPolavarapu, LakshminarayanaDobrowolski, PiotrPolicicchio, AlfonsoDobrzynski, PiotrPolimeni, AntonioDocea, AncaPolini, RiccardoDocoslis, ArisPolitano, AntonioDolado, Jorge S.Polito, LauraDolganov, Pavel VladimirovichPollice, AlessandraDolocan, AndreiPollmann, KatrinDomine, Marcelo E.Polo, FedericoDomingues, Eddy M.Polo-Parada, LuisDominguez-Pumar, ManuelPolosan, SilviuDominguez-Robles, JuanPoma, AnnaDonato, Ricardo KeitelPomastowski, Paweł P.Dong, JunhangPomposo, José A.Dong, QiuchenPonomarev, Igor I.Dong, RenhaoPons, MichelDongil, Ana B.Pons, RamonDonnadio, AnnaPons, ThomasDonnelly, RyanPonterio, Rosina CelesteDonzelli, ElisabettaPoodt, PaulDoranehgard, Mohammad HosseinPopa, LacramioaraDorao, Carlos A.Popat, KetulDordevic, Sasa V.Pope, MichaelDoris, EricPopescu, Carmen-MihaelaDorman, JamesPopescu, Ionel CatalinDorner-Reisel, AnnettPopescu, Mihail N.Dorofeenko, Alexander V.Popescu, RadianDorosz, DominikPoplawska, MagdalenaDorozhkin, Sergey V.Popov, CyrilDory, DanielPopova, MargaritaDoustkhah Heragh, EsmaeilPorcel, ErikaDoustkhah, EsmailPorrati, FabrizioDouvris, ChrisPortugal, Camila C.Downing, CharlesPosselt, DortheDracopoulos, VassiliosPostigo, Pablo AitorDrăgoi, Cristina ManuelaPostnikov, Pavel S.Dragoman, DanielaPoteser, MichaelDragoman, MirceaPotrich, CristinaDragos, HorvathPou, JuanDragutan, ValerianPourrahimi, Amir MasoudDrazic, GoranPourroy, GenevieveDreizin, Edward L.Pozdnyakov, Ivan P.Dressel, MartinPrabhakaran, VenkateshkumarDrevet, RichardPrabhu, Subbaiah MuthuDrillet, Jean-FrançoisPradhan, Dhiren K.Driussi, FrancescoPrado-Gonjal, JesúsDrobne, DamjanaPrado-Gotor, RafaelDrongelen, Martin VanPramanik, ArindamDrozdov, Aleksey D.Pramanik, AvijitDu, ShangfengPrashanth, Konda GokuldossDu, XushengPrassl, RuthDuan, WeiPrat, MariaDubowik, JanuszPrati, CarloDuboz, Jean-YvesPrati, FrancoDubrovskii, VladimirPratsinis, HarrisDuchene, JosephPraus, PetrDudem, BhaskarPravica, Michael G.Dudina, Dina V.Preda, NicoletaDudziec, BeataPredoi, DanielaDueñas Carazo, SalvadorPricl, SabrinaDuerkop, AxelPrida, Victor M.Dughiero, FabrizioPrieto, CristinaDulski, MateuszPrieto, Miguel A.Dummer, Nicholas F.Prikulis, JurisDumur, FrédéricPristovsek, MarkusDuncan, Timothy V.Privitera, Stefania M.S.Dunyushkina, LiliyaPrlić Kardum, JasnaDuong, Dinh LocProchazka, MarekDurajski, Artur P.Prochowicz, DanielDurán-Valle, Carlos J.Prost, WernerDurazzo, AlessandraProtesescu, LoredanaDurek, ThomasProust, JulienDurndell, LeeProverbio, EdoardoDusinska, MariaPruna, AlinaDušková-Smrčková, MiroslavaPruna, Alina IulianaDuta, LiviuPruneanu, Stela-MariaDuval, Raphaël E.Prusik, KrystianDuzgunes, NejatPryamikov, AndreyDvoranova, DanaPtaszek, MarcinDyakonov, Grigory S.Ptok, AndrzejDybko, KrzysztofPu, Ying-ChihDybtsev, DanilPucci, AndreaDzantiev, BorisPuértolas, Jósé AntonioDzhardimalieva, GulzhianPuglia, DeboraDziedzic, AndrzejPuglisi, RosariaDziubek, KamilPui, AurelEbara, MitsuhiroPuigdollers, JoaquimEbert, Timothy APuigmartí-Luis, JosepEbralidze, Iraklii I.Pulgar, VictorEckschlager, TomasPulham, ColinEdgar, James H.Puli, Venkata SreenivasEdirisinghe, MohanPulidindi, Indra NeelEdlund, UlricaPulido-Melián, ElisendaEersels, KasperPunta, CarloEfimov, AlexanderPuosi, FrancescoEftekhari, AliPurcar, VioletaEgarievwe, Stephen U.Purcell-Milton, FinnEgberts, PhilipPuri, AnuEhrlich, HermannPushkar, YuliaEhrmann, AndreaPussi, KatariinaEimutis, JuzeliūnasPuszkiel, Julián A.Eklund, PerPutkonen, Matti Il­mariEl Euch, HichemPutrinš, MartaEl Kadib, AbdelkrimPutz, Mihai V.El Mehtedi, MohamedPyatenko, AlexanderEl Yamani, NaoualePyrgioti, EleftheriaElaissari, AbdelhamidQi, CongElbuken, CaglarQi, DongchenElemans, Johannes A.A.W.Qiao, RuiruiEleni, MakaronaQiao, YuEleonora, RussoQiu, GangEleuch, HichemQuadir, MohiuddinEl-Gendy, Ahmed A.Quarta, AlessandraEliseeva, Svetlana V.Quax, PaulEljamal, OsamaQuesne, MatthewEl-Kirat-Chatel, SofianeQuijada-Garrido, IsabelEllahi, RehmatQuiles, FabienneEllinas, KosmasQuintanilla, AsuncionElmquist, Randolph E.Quochi, FrancescoEl-Ramady, HassanQuynh Hoa, LeEl-Safty, SherifRaabe, GabrieleEmadi, ArezooRabanal, María EugeniaEmeline, AlexeiRabczuk, TimonEmelyanov, Andrey V.Rabilloud, ThierryEndalkachew, Sahle-DemessieRabouw, Freddy T.Endoh, TamakiRabu, PierreEnesca, AlexandruRachwalski, MichałEngelmann, PeterRacuciu, MihaelaEnglish, NiallRaczkowska, JoannaEnnen, IngaRadamson, Henry H.Enríquez Pérez, EstherRadaram, BhaskerEnyashin, AndreyRadenković, SlavkoEpifani, MauroRadu, MihaiEriksson, JensRadulescu, CristianaEritja, RamonRadulovic, JovanaErwan, PaineauRadziuk, DaryaEscapa, AdrianRaemdonck, KoenEscoda, LluïsaRafael, LuqueEscola, José MaríaRafhay, QuentinEslami, HosseinRafiee, MohammadEsmaeely Neisiany, RasoulRafique, SubrinaEspinosa, AnaRaftopoulos, KonstantinosEspita, PaulaRagab, TarekEsposito Corcione, CarolaRaganati, FedericaEsposito, GennaroRagona, LauraEsposito, SerenaRagusa, AndreaEspuelas, SocorroRahimi-Gorji, MohammadEsquivel-Upshaw, Josephine F.Rahman Rashid, Rizwan AbdulEs-Souni, MohammedRahman, Md. MahbuburEstel, LionelRahme, KamilEsteves Da Silva, Joaquim C.G.Raica, MariusEstrader, MartaRaikher, Yuriy L.Estrany, FrancescRaimondo, MarialuigiaEtacheri, VinodkumarRaimondo, MariarosaEuchner, HolgerRainer, AlbertoEudald, CasalsRaj, EldonEvangelisti, ClaudioRajagopal, SudarshanEvans, DrewRajagopalan, Uma MaheswariEven-Hernandez, PascaleRajashekara, GireeshEvtugyn, GennadyRakhubovsky, Andrey A.Ewald, AndreaRakoczy, RafalEzhov, MaratRalchenko, VictorEzquerra, TiberioRamadoss, AnanthakumarFabbri, FilippoRamakumar, KinthadaFabbri, PaolaRamapuram, JayFábián, IstvánRamazani, AliFabrizio, FabrizioRamezani, ZeinabFagadar-Cosma, EugeniaRamiasa, MelanieFaggio, GiulianaRamírez, MartínFairclough, SimonRamirez-Castellanos, JulioFakhrullin, Rawil F.Ramirez-Vick, JaimeFakis, MihalisRamis, GianguidoFalciani, ChiaraRamos Cabrer, PedroFalcieri, ElisabettaRamos Loja, Maria AméliaFalcó, AlbertoRamtoola, ZebunnissaFalk, Abram L.Ranc, VáclavFalk, MartinRancan, FiorenzaFalletta, ErmelindaRangari, VijayFam, Tkhe KyongRanieri, AntonioFanciulli, MarcoRanoszek-Soliwoda, KatarzynaFang, QingmingRanzato, EliaFangueiro, JoanaRao, RahulFanizzi, Francesco PaoloRaposo, MariaFaraldos, MarisolRapp, LudovicFarcas, MarianaRascio, AgataFarcau, CosminRashid, Mohammad MamunurFarè, SilviaRashidi, Mohammad MehdiFarhat, SamirRasskazov, IliaFarjas, JordiRastogi, AlokFarka, ZdeněkRathousky, JiriFarnood, Ramin R.Ratnakumar, Bugga V.Farrow, Reginald C.Raty, Jean-Yves RatyFateixa, SaraRätzke, KlausFatyeyeva, KaterynaRatzke, MarkusFavaro, MarcoRaucci, Maria GraziaFavier, LidiaRaudino, AntonioFavre-Réguillon, AlainRauschenbach, BerndFazio, BarbaraRauwel, ErwanFdez-Gubieda, Maria LuisaRauwel, ProtimaFebvre, PascalRavelli, DavideFedele, FrancescoRavi Kumar, Majeti N. V.Fedele, LauraRavindra, NuggehalliFedele, MonicaRavnik, JureFedorov, GeorgyRavula, SudhirFedorov, VladimirRaza, AunFedriani, Luis EsquiviasReber, ChristianFeith, DavidRebholz, ClausFejfar, AntonínRebollar, EstherFeldblyum, Jeremy I.Rebrov, EvgenyFelgueiras, HelenaRecher, PatrikFemoni, CristinaRecio, José ManuelFeng, TianliReddy, M. Siva PratapFerella, FrancescoRees, Andrew S.Ferhan, Abdul RahimReglero Ruiz, José AntonioFernandes, AntonyRehder, DieterFernandes, AugusteRehm, Thomas H.Fernandes, Susete NogueiraReid, Michael S.Fernández Ramos, María DoloresReid, ScottFernández, AdolfoReineck, PhilippFernandez, CarlosReinosa, Julián JiménezFernández, IgnacioReipa, VytasFernandez, InmaculadaReis, Glaydson Simoes DosFernández, Juan PedroRej, SouravFernández-Arévalo, MercedesRejinold, SanojFernández-García, MartaRekas, MieczyslawFernández-Lázaro, FernandoRella, RobertoFernández-Pradas, J.M.Remedios Serrano, DoloresFernández-Romero, Juan ManuelRene, EldonFerrando Soria, JesúsRener-Sitar, KsenijaFerrando, RiccardoRenge, IndrekFerranti, PasqualeRenger, ThomasFerrari, ClaudioRengo, SandroFerrari, ErikaRenu, SankarFerrari, LucRenz, MichaelFerraris, SaraReschetilowski , WladimirFerraro, DarioResmini, MarinaFerraro, GiaritaRevnic, CorneliaFerraz, RicardoRey, AlejandroFerré-Borrull, JosepRey, AnaFerreira, HelenaReyes-Ortega, FelisaFerreira, PaulaReza, Khan MamunFerrer, BelenRhee, Jin-KyuFerretti, Anna MariaRhee, Jong IlFerrier, LydieRho, JunsukFey, TobiasRhyee, Jong-sooFiandra, LuisaRibeiro, DanielaFic, KrzysztofRicci, MarilenaFicek, MateuszRicci, Pier CarloFierascu, Radu ClaudiuRiccò, RaffaeleFierro, VanessaRichard, Caroline S.Fifere, AdrianRichard, CyrilleFigueira, FlavioRichards, ChrisFigueiras, AnaRichardson, JosephFigueiredo, JanineRichter, Günther H.S.Filgueira, CarlyRichter, KatharinaFilice, MarcoRichtera, LukášFilip, AdrianaRico, Victor J.Filip, PetrRicote, SandrineFilipescu, MihaelaRider, Andrew N.Filippini, TommasoRigutti, LorenzoFilová, EvaRiha, Shannon C.Fina, IgnasiRimpelová, SilvieFioravanti, GiuliaRinaldi, DanieleFiori, FabrizioRincon Joya, MiryamFiorilli, SoniaRinkevich, AnatolyFiorillo, AntoninoRinnerthaler, MarkFiorito, SilvanaRíos, Ángel ÁFirdessa, RebumaRipolles, Teresa S.Fischer, DagmarRishinaramangalam, AshwinFischer, Hartmut R.Rishpon, JudithFischer, HolgerRistic, AlenkaFischer, Inga AnitaRistori, SandraFischetti, Massimo V.Rivadeneyra, AlmudenaFisher, BertinaRivero-Müller, AdolfoFisher, John G.Rives, VicenteFistul, MikhailRizal, ConradFitta, MagdalenaRiziotis, ChristosFitzgerald, Stephen A.Rizza, GiancarloFlandin, LionelRizzarelli, PaolaFleaca, C.T.Rizzi, Gian AndreaFletcher, NicholasRizzolio, FlavioFleury, EricRo, InsooFlieger, JolantaRoberto Giannuzzi, RobertoFlorczyk, StephanieRobichaud, DavidFlorea, AdrianRocco, LuciaFlorea, IleanaRoddaro, StefanoFlores, Ana IsabelRodrigues, FranciscaFlores-Colen, InêsRodríguez Pascual, AlejandroFlorin, Marinca TraianRodriguez, AngelFlorio, GiuseppeRodríguez, FernandoFlox, CristinaRodriguez, LuisFodor, FerencRodriguez, NoelFoelske, AnnetteRodríguez, RafaelFois, EttoreRodríguez-Argüelles, María CarmenFollink, BartRodriguez-Lorenzo, LauraFologea, DanielRodriguez-Seijo, AndresFominski, V. YuRogl, GerdaFong, Wye-KhayRoh, ChanghyunFonin, MikhailRoh, Jong WookFontana, AntonellaRoh, Kyung-HoFontana, FlaviaRoig, AnnaFontanesi, ClaudioRojas-Hernandez, Rocío EstefaníaFonteriz, Rosalba I.Rojo, JoséFoo, SimonRokosz, KrzysztofFoot, PeterRoldán, Juan BautistaForay, GenevieveRolfe, PeterForgan, RossRollini, ManuelaFormela, KrzysztofRomanato, FilippoFornasiero, PaoloRomán-Martínez, M. CarmenForrer, DanielRomano, LuciaFort, AdaRoma-Rodrigues, CatarinaFortunati, IlariaRomeira, BrunoForzato, CristinaRomero, IsabelFountos, GeorgeRomero-Salguero, Francisco J.Fragoso, AlexRosa, LorenzoFraix, AuroreRosenkranz, AndreasFrances Monllor, JorgeRosenzweig, DerekFrancesca, SelminRosi, MarzioFrancesco Sgroi, MauroRosini, ElenaFranckevičius, MariusRoslova, MariaFranco, Camilo AndresRossella, FrancescoFrançois Fréty, Roger ThomasRossetti, IleniaFrank, IrmgardRossi, CaroleFrankcombe, Terry J.Rossi, ClaudioFrank-Kamenetskaya, Olga V.Rossi, LorenzoFranus, WojciechRossi, MarcoFratila, RalucaRossi, StefanoFrattini, DomenicoRossner, PavelFrazer, LaszloRostovtsev, YuriFreakley, SimonRoszak, SzczepanFresch, BarbaraRotaru, AndreiFreyria, FrancescaRoth, Stephan V.Freyria, Francesca StefaniaRothbauer, MarioFriák, MartinRougier, EstebanFrielinghaus, HenrichRousseau, RogerFrigerio, JacopoRoussey, MatthieuFrogley, MarkRoussis, Ioannis G.Fröhlich, EleonoreRovida, CostanzaFrontera, AntonioRowe, AlistairFruth, Victor OprisanRoy, RenèFu, GengtaoRoy, SagarFu, KunRozhkova, NataliaFu, LiansheRozynek, ZbigniewFucikova, AnnaRubini, SilviaFudala, RafalRubino, Federico MariaFudouzi, HiroshiRuda (FRSC), HarryFugetsu, BunshiRudiuk, SergiiFuguet, ElisabetRudolf, RebekaFuh, Lih-JyhRudshteyn, BenjaminFujii, TakenoriRuelland, EricFujiki, MichiyaRuello, PascalFujiwara, AkihikoRuffino, FrancescoFukuda, NobukoRuffolo, SilvestroFukuda, TakeshiRui, YukuiFukushima, TomohiroRui, ZhenhuaFulvio, Pasquale F.Ruiz De Ballesteros, OddaFulvio, Pasquale FernandoRuiz-Rosas, Ramiro RafaelFurdyna, JacekRumyantseva, MarinaFurumi, SeiichiRunowski, MarcinFurusawa, HiroyukiRurali, RiccardoFuruta, MamoruRusciano, GiuliaFuso, FrancescoRussell, Steve T.Futaba, Don NorimiRusso Krauss, IreneFutakuchi, MitsuruRusso, AlessandroFydrych, DariuszRusso, VincenzoGaan, SabyasachiRusu, DanielaGabay, Alexander M.Rüter, Christian E.Gábor, KatonaRüther, ThomasGaczynska, MariaRutkowska, MałgorzataGadisa, Abay DinkuRuysschaert, Jean MarieGadomski, AdamRyabchikov, YuryGaffet, EricRyabikin, Mikhail Yu.Gaipl, Udo S.Ryazanov, ValeryGalán, Miguel LaderoRydosz, ArturGalian, Raquel E.Ryu, Ja-HyoungGalinetto, PietroRyu, JehwangGallavardin, ThibaultRyu, Seung YoonGallei, MarkusRzeźnicka, Izabela I.Gallo, JuanRzymski, PiotrGallo, MonicaSá, JacintoGallo, SalvatoreSá, RosáliaGallo, SimoneSaasen, ArildGalstyan, VardanSabatini, LuigiaGalvanetto, EmanueleSacchetti, FrancescoGalzitskaya, Oxana V.Saccomandi, PaolaGambardella, AlessandroSachdeva, Ir. SumitGamella Carballo, MariaSacher, EdwardGamulin, OzrenSachse, AlexanderGan, Chee KwanSadovnikov, AlexandrGanazzoli, FabioSadowska, BeataGanikhanov, FeruzSadowska, KamilaGannett, Peter M.Saez, CristinaGanta, DeepakSafaei, Mohammad RezaGao, DunfengSager, ManfredGao, JinSaghir, ZiadGao, NanSagué, Anna LaromaineGao, ZheSaha, Jhantu KumarGaponik, NikolaiSaha, Manik LalGarbovskiy, YuriySahni, Sanjeev K.Garbuzenko, OlgaSahoo, SanjubalaGarcia Amorós, JaumeSahraoui, BouchtaGarcía Mandayo, GemmaSahu, Saura C.García Ruiz, Francisco JavierSaielli, GiacomoGarcía Sancho, CristinaSaini, NaurangGarcia, Andres SanzSaito, GenkiGarcia, Coralia V.Saito, MasatoGarcía, GregorioSaito, NorikoGarcía, HéctorSaito, YutaGarcia, Maria Isabel DiezSajgalik, PavolGarcía, Miguel TorresSajkiewicz, PawełGarcía, NuriaSakaebe, HikariGarcia-Benett, AlfonsoSakai, JoeGarcia-Deibe, AnaSakai, ShigekiGarcía-Fresnadillo, DavidSakakura, MasaakiGarcia-Fuentes, MarcosSakar, MohanGarcia-Gallego, SandraSakthivel, Tamil SelvanGarcia-Garcia, Francisco J.Saladino, Maria LuisaGarcia-Gonzales, CarlosSalado, ManuelGarcía-Lastra, Juan MaríaSalak, AndreiGarcía-Martinez, Joaquín C.Salamin, YannickGarcía-Miquel, HectorSalaoru, IuliaGarcia-Pomar, Juan LuisSalari, AlinaghiGarcía-Rupérez, JaimeSaleem, ImranGarcia-Segura, SergiSalem, BassemGarcia-Verdugo, IgnacioSalerno, MarcoGardikis, KonstantinosSalim, MalindaGardiner, PhilipSalinas-Castillo, AlfonsoGargiulo, ValentinaSalis, AndreaGariglio, StefanoŠalkus, TomasGarino, NadiaSalles, FabriceGarmanchuk, Liudmyla V.Saltas, VassiliosGaroli, DenisSalvato, MatteoGarriga, RosaSalvatori, StefanoGarro, NúriaSalvi, Anna M.Garroni, SebastianoSalzillo, TommasoGarten, LaurenSamad, Yarjan AbdulGartia, Manas RanjanSamaras, IoannisGary-Bobo, MagaliSambur, Justin B.Gas, PiotrSamhan-Arias, AlejandroGashti, Mazeyar ParvinzadehSamiei, EhsanGaskov, Alexander M.Samoila, P. MugurelGaspar, Maria ManuelaSamokhin, Andrey V.Gaspar, VítorSan Andrés, María PazGastaldi, GiuliaSan Miguel, VeronicaGatica, José M.Sanchez, GoarGatin, AndreySánchez-García, DavidGatti, AntoniettaSánchez-Royo, Juan FranciscoGatti, TeresaSánchez-Sánchez, ManuelGatto, EmanuelaSancini, GiulioGatto, IreneSandanayaka, Atula S.D.Gauden, PiotrSanderson, JosephGautam, Siddharth S.Sandu, TitusGautier, GaelSandu, ViorelGautier, MathieuSangiovanni, DavideGaviña, PabloSankaran, Kamatchi JothiramalingamGavini, ElisabettaSannino, DianaGavrilov, SergeySannino, FilomenaGavrilova, NataliaSanson, AndreaGaynor, WhitneySansone, AnnaGazda, MariaSantaballa, J. ArturoGebicki, JacekSantagata, AntonioGedvilas, MindaugasSanthanam, KSVGeffroy, BernardSantini, AntonelloGeissler, DanielSantos, AbelGelbstein, YanivSantos, ElizabethGeletii, YuriiSantos, MiguelGellini, CristinaSantos-Juanes, LucasGenchi, GiadaSantos-Ruiz, LeonorGenerosi, AmandaSantschi, ChristianGenninati Crich, SimonettaSañudo, E. CarolinaGenorio, BostjanSanz Ortiz, Ángel SantiagoGenova, TullioSapi, AndrasGenovese, DamianoSarafraz, Mohammad MohsenGentile, GennaroSarantopoulou, EvangeliaGentile, PietroSarasua, Jose-RamonGentili, DenisSaravana, Sivagnanam PeriaswamyGentilucci, LucaSaravanakumar, KandasamyGeorgakilas, AlexandrosSarbu, AndreiGeorgakilas, VasiliosSaremi, MehdiGeorge, Thomas F.Šarić, AnkicaGeorgieva, Jenia S.Sarkar, TapatiGeorgii, RobertSarker, DipakGeorgios, KopidakisSarris, IoannisGeraldes, CarlosSarris, Ioannis E.Geremia, SilvanoSarswat, Prashant K.Gerislioglu, BurakSarvestani, AlirezaGeso, MoshiŠatínský, DaliborGesuele, FeliceSato, HideyukiGeszke-Moritz, MałgorzataSato, KazunoriGetautis, VytautasSato, KimiyasuGhamouss, FouadSato, Shunsuke A.Ghanekar, AlokSato, YusukeGhanotakis, Demetrios F.Satoh, ShigeruGhazzal, Mohamed NawfalSatriano, CristinaGheju, MariusSavateev, AleksandrGhenuche, PetruSavilov, SergueiGheorghiu, MihaelaSawada, ToshikiGherardi, FrancescaSawicki, MaciejGherardi, MatteoSayed, MurtazaGhica, DanielaSayyafzadeh, MohammadGhica, Mariana EmiliaScaffaro, RobertoGhidelli, MatteoScaini, DenisGhodake, GajananScala, AngelaGhorbani-Asl, MahdiScalese, SilviaGhosh, DebanjanaScamarcio, GaetanoGhosh, SubrataScandurra, AntoninoGhoshal, SushantaScandurra, RobertoGhoshal, TandraScarano, AntonioGiacomello, AlbertoScardaci, VittorioGiancane, GabrieleScarfato, PaolaGiannakoudakis, Dimitrios A.Scarselli, ManuelaGiannazzo, FilippoSchaefer, Hans-EckhardtGiannini, VincenzoSchäfer, HelmutGiannone, GrégoryScheer, Hella-ChristinGiebeler, LarsScheiner, SteveGigliotti, Casimiro LucaSchepler, KennethGil, BarbaraScherthan, HarryGillan, Edward G.Schiek, ManuelaGille, LarsSchierbaum, Klaus DieterGil-Ramirez, GuzmanSchift, HelmutGinovska, BojanaSchilirò, EmanuelaGiorcelli, MauroSchintke, SilviaGiordano, Maria CaterinaSchiraldi, David A.Giordano, StefanoSchlettwein, DerckGiorgetti, LuciaSchmedake, Thomas A.Giorgio, IvanSchmelzer, Jürn W.P.Giovagnoli, StefanoSchnabel, ManuelGiovannetti, RitaSchneider, Hans ChristianGirolami, MarcoSchneider, NathanaelleGîrțu, Mihai A.Schneider, RaphaëlGitsas, AntonisSchneider, Yves-JacquesGiunchedi, PaoloSchnell, Sondre K.Glavina, DomagojSchniepp, HannesGliemann, HartmutScholz, FerdinandGlomm, Wilhelm R.Scholz, MiklasGłuchowski, PawełSchowalter, MarcoGlukhova, Olga E.Schrefler, BernhardGlüsen, AndreasSchröder, SvenGnade, Bruce E.Schröder, ThiesGnyba, MarcinSchroeder, Marshall A.Gobeze, HabtomSchulze-Tanzil, Gundula GesineGodinho, Maria HelenaSciacca, Michele Francesco MariaGodino-Salido, Maria LuzScicchitano, PietroGodlewski, MarekSciortino, AliceGolding, JonScirè, SalvatoreGoldoni, MatteoScitharan, ThirumaneGolewski, PrzemysławScognamiglio, VivianaGolub, IgorScotognella, FrancescoGombart, AdrianScribante, AndreaGómez Polo, CristinaScuderi, VivianaGómez-Cámer, Juan LuisSebastián, DavidGómez-Graña, SergioSebastian, VictorGómez-Herrero, JúlioSecenji, AleksandarGomonay, OlenaSecu, Elisabeta-CorinaGömöry, FedorSecu, MihailGonçalves, Helena M. R.Sedlačík, MichalGonçalves, LídiaSegawa, HiroyoGonçalves, RenatoSeguin, Jean LucGong, MaogangSegura, Miguel F.Goni, FelixSeibt, MichaelGonzález, BegoñaSeifert, GotthardGonzalez, JoaquinSeifitokaldani, AliGonzález, SergioSeijas , Julio A.Gonzalez, ZoraidaSeisenbaeva, GulaimGonzález-Diaz, Óscar M.Seitz, William RudolfGonzález-García, CristinaSekatski, SergueiGonzález-Minero, Francisco JoséSeko, NoriakiGonzalez-Pedro, VictoriaSel, OzlemGonzalez-Platas, JavierSelim, FaridaGopalakrishnan, KothandamSelke, MatthiasGopalan, SaianandSellars, JonathanGora-Marek, KingaSellier, MathieuGoraus, JerzySelopal, Gurpreet SinghGordeev, SergeySemaltianos, Nikolaos G.Gordon, DanielSemancik, StephenGoreham, ReneeSemenov, KonstantinGorgieva, SelestinaSementa, LucaGorin, DmitrySemisalova, AnnaGorji, Nima E.Senel, MehmetGorte, Raymond J.Seo, ChulhunGorzelanny, ChristianSeo, Dae-ShikGośliński, TomaszSeo, SoonminGoswami, NirmalSeo, SungbaekGotić, MarijanSeo, Young-KwonGoto, YosukeSeol, Myeong-LokGottfried, JenniferSeong, GimyeongGottwald, AlexanderSequeira, César A. C.Gourmans, Marie-JoséSerafín, VeronicaGovan, JosephSerda, RitaGracin, DavorSerpe, LoredanaGradov, Oleg M.Serpooshan, VahidGradov, Oleg V.Serrà, AlbertGraf, WilhelmSerrano, AidaGrande Tovar, Carlos DavidSerrano, Juan CarlosGrande, MarcoSeto, YoshikiGrange, RachelSevcsik, EvaGranitzer, PetraSeveyrat, LaurenceGranozzi, GaetanoSevilla, MichaelGrasset, FabienSevilla-Morán, BeatrizGravalidis, ChristoforosSeydou, MahamadouGravel, EdmondSgarbossa, PaoloGraves, JosephSgarzi, MassimoGraziani, GabrielaShabatina, TatyanaGrebel, HaimShacham-Diamand, YosiGreco, AntonioShadike, ZulipiyaGreco, GiorgiaShadloo, Mostafa SafdariGreczynski, GrzegorzShafirovich, EvgenyGregorcic, PeterShah, Nilam C.Grenzer, JörgShakhaoath, Md.Grieb, PawelShameer, KhaderGries, ThomasShamoto, ShinichiGrigorova, EliShan, ShiyaoGrigsby, Christopher L.Shankar, EswarGrijalvo, SantiagoShao, LeiGrilc, MihaShapter, JoeGrivel, Jean-ClaudeSharker, Shazid Md.Grodzik, MartaSharma, KavitaGrolli, StefanoSharma, PankajGromov, Alexander A.Sharma, Priyanka R.Grossi, MarcoSharma, Sanjeev K.Grosu, YaroslavSharma, Shiv K.Groza, AndreeaShaula, AliaksandrGrumezescu, Alexandru MihaiShaulsky, EvyatarGrunwald, RuedigerShavandi, AminGryglewicz, GrazynaShcharbin, DzmitryGrzelczak, MarekSheardy, RichardGualdrón-Reyes, Andrés-FabiánShearer, CameronGuan, XiaoweiSheka, Elena F.Guardia, PabloShellaiah, MuthaiahGuascito, Maria RacheleShelushinina, Nina G.Gubala, VladimirShen, XiGude, Veera GnaneswarShenderovich, Ilya G.Gudkov, Sergey V.Sheremet, MikhailGudmundsson, Jon TomasShevelkov, Andrei V.Güemes, AlfredoShi, KaiyuanGuénin, ErwannShibahara, MakotoGueorguiev, Gueorgui K.Shibata, HiroyukiGuerin, KatiaShiddiky, MuhammadGuerrero, AntonioShie, Ming-YouGuerrero-Pérez, M. OlgaShieh, Dar-BinGuerrieri, AntonioShieh, Jia-MinGuidi, PatriziaShih, Shao-juGuidotti, MatteoShiku, HitoshiGuillard, ChantalShim, Jun HoGulari, ErdoganShimada, ToshihiroGuldiken, RasimShimakawa, KoichiGulino, AntoninoShimanouchi, ToshinoriGun’ko, YuriiShimizu, HiromasaGuney, DurduShimizu, JunGunnar, KristoferShimomura, TakeshiGuo, Zhan-HuShin, Bo-SungGuo, ZhinanShin, Weon HoGupta, ChiragShinde, Surendra KrushnaGupta, MohitShiomori, KoichiroGupta, SanjuShiratani, MasaharuGupta, VijaiShirokov, Vladimir B.Gurevich, Evgeny L.Shironita, SayokoGurevich, LeonidShirvanimoghaddam, KamyarGurioli, MassimoShiue, Ren-KaeGurzhiy, Vladislav V.Shiver, LembitGusev, AlexanderShokouhimehr, MohammadrezaGusiatin, Zygmunt MariuszShorstkii, IvanGuskos, NikoShorubalko, IvanGusmão, RuiShoseyov, OdedGutierrez, JonShoute, Lian C.T.Gutsch, SebastianShpacovitch, VictoriaGutzov, StoyanShrestha, LokGuzmán, EduardoShrestha, ManiGuzman, MarceloShrestha, Nabeen K.Gyergyek, SašoShtepliuk, IvanGyurcsik, BelaShukla, SourabhHa, Don-HyungShvartsman, VladimirHabib, KhaledSibirev, NickolayHackenberg, StephanSicard-Roselli, CécileHaddadi, AbbasSidorenko, AlexanderHadjadj, AomarSiegel, JakubHadjiargyrou, MichaelSiegel, JanHadjiev, Viktor G.Šiffalović, PeterHadjikakou, Sotiris K.Sigle, WilfriedHadjipanayis, George C.Signore, GiovanniHagita, KatsumiSigurjonnson, OlafurHaguet, VincentSikavitsas, VassiliosHahn, Jae RyangSikiric, MajaHai, Pham NamSikora, AndrzejHai, ZhenyinSikora, MarcinHakuta, YukiyaSikora, PawełHalappanavar, SabinaSikorski, AleksanderHalbritter, AndrásSilien, ChristopheHall, ColinSilikas, NikolaosHall, SteveSiliqi, DritanHallam, TobySillerud, LaurelHallén, AndersSilva, Ana RosaHaluska, MiroslavSilva, CarlaHamad-Schifferli, KimberleySilva, Maria JoaoHamidi-Asl, EzatSilva, Maria ManuelaHamilton, JeremySilvan, Miguel MansoHamon, MorganSilveira, CéliaHampel, SilkeSilvestre, SamuelHampp, Norbert A.Silvestri, BrigidaHan, Dong-PyoSimanjuntak, Firman MangasaHan, Dong-WookSimeone, PasqualeHan, Gill SangSimion, CristianHan, Jun HyunSimion, Cristian E.Han, MeikangSimka, WojciechHan, QianSimoen, EddyHan, Youngkyu Simões, SóniaHanaor, Dorian A.H.Simon, JohnHandke, BartoszSIMONATO, Jean-PierreHanif, AsadSimončič, BarbaraHaniu, HisaoSimonescu, Claudia MariaHansma, HelenSimonič, MarjanaHao, NanjingSimserides, ConstantinosHao, XiaojuanSimula, AlexandreHaponiuk, JózefSingh, Ajay VikramHara, MasanoriSingh, DavidHaraguchi, NaokiSingh, Dharmendra PratapHardy, John GeorgeSingh, JaiHariyama, TakahikoSingh, NeenuHarja, MariaSingh, Ravi NandanHärk, EneliSingh, SubhashHarker, AnthonySinha Ray, SaikatHarmandaris, Vagelis A.Sinha Ray, SumitHarničárová, MartaSinha, RajuHarnois, MaximeSinha, Sudarson SekharHarris, David T.Sinha-Ray, SumanHarrison, Roger G.Siniscalco, DarioHartings, Matthew R.Sinn, GerhardHartnagel, HansSipes, BrentHartwig, AndreaSipos, PálHasegawa, GeorgeSironi, LauraHasegawa, MikiŠírová, MiladaHashishin, TakeshiŠišková, Karolína MachalováHassan, Sammer-ulSitarz, MaciejHatakeyama, KazutoSiuzdak, KatarzynaHatamoto, MasushiSivaramapanicker, SreejithHattar, KhalidSiwek, AgataHattori, Azusa N.Siwińska-Ciesielczyk, KatarzynaHattori, Haroldo T.Sizov, Fedir FedorovychHatzikraniotis, EvripidisSkapin, Sreco D.Haugen, Håvard J.Skeldon, PeterHauser, AndreasSkirtach, AndreHawash, ZaferSkládal, PetrHay, SamSkoko, ŽeljkoHayakawa, TohruSkrifvars, MikaelHayashi, KatsuroSkwarek, EwaHayashi, YoheiSlabon, AdamHe, GuanjieSlama, JozefHe, JianŚlebarski, AndrzejHe, JiaruiSleigh, AlisonHe, JieSlepicka, PetrHe, QiyuanSloan, JeremyHe, XinSlouka, ZdenekHe, YurongSluimer, JudithHebrant, MarcSmani, YounesHector, MarkSmirnov, Alex I.Hedberg, JonasSmirnov, VladimirHeddle, Jonathan G.Smith, Adam E.Hefferon, Kathleen LauraSmith, Edward R.Hegab, HussienSmith, PaulHeger, ZbynekSmith, Paul F.Heiderhoff, RalfSmith, Thomas J.Heier, JakobSmolin, AlexeyHeimdal Nilsson, ElnaSmulko, JanuszHejna, AleksanderSnawder, JohnHémadi, MiryanaSnee, PrestonHempel, MartinSnegur, LubovHendon, ChristopherSoave, RaffaellaHeng, Lee KhengŠob, MojmírHenych, JiříSobisch, TitusHepel, MariaSobolev, KonstantinHeras, AránzazuSobolewski, PeterHerckes, PierreSobota, Radoslaw M.Hermenean, AncaSobotta, ŁukaszHernández Creus, AlbertoSocas-Rodríguez, BárbaraHerranz, FernandoSocol, MarcelaHerranz, Maria AngelesSödergård, AndersHerten, MonikaSogorb Sánchez, Miguel ÁngelHesp, Simon A.M.Sohn, Jung WooHess-Dunning, AllisonSohn, SungWooHettegger, HubertSohn, YoungkuHeuer-Jungemann, AmelieSójka, LukaszHeuken, MichaelSoldano, CaterinaHeyda, JanSolin, NiclasHeydari, GisooSologubenko, AllaHeyn, ChristianSolorio, LuisHickerson, RobynSolovieva, AnastasiyaHigaki, TatsuyaSom, ClaudiaHijikata, YasutoSon, Seung UkHill, JonathanSone, HidekoHill, JosephineSong, Ho JunHilleringmann, UlrichSong, Im SookHinderliter, BrianSong, KenanHippler, RainerSong, Nam WoongHirscher, MichaelSong, SunghoHirva, PipsaSong, Tze-BinHisada, KenjiSong, Young MinHjelme, Dag RoarSong, ZiyuanHng, Huey HoonSoria Sanchez, MariaHnida, KatarzynaSotgiu, GiovannaHo, Chia-CheSotgiu, GiovanniHo, DanielSotiropoulos, SotirisHo, Yi-ChengSoto, FernandoHobbie, Erik K.Soto, PaulHoffmann, Markus M.Sotta, PaulHogan, James D.Soveral, GraçaHogenEsch, HarmSpadavecchia, JolandaHojo, DaisukeSpagnolo, BernardoHolefors, AnnaSpagnuolo, CarmelaHoller, StephenSpalek, JozefHolmberg, VincentSpanopoulos, IoannisHolst, BodilSpasova, MariyaHolynska, MalgorzataSpeghini, AdolfoHolze, RudolfSpeliotis, ThanassisHomaeigohar, ShahinSperanza, GiorgioHoncová, PavlaSpirk, StefanHong, HaipingSpitalsky, ZdenoHong, Jeong HeeSportelli, Maria ChiaraHong, MinghuiSprio, SimoneHong, Suck WonSprynskyy, MyroslavHong, SukjoonSrdic´, VladimirHooper, Justin B.Sreekanth, Kandammathe V.Hopkinson, MarkSrinivasa Rao, SunkaraHöppener, StephanieSrinivasan, Dinesh KumarHorchani, HabibSriram, GopuHornebecq, VirginieSrivastava, Gyaneshwar P.Hörner, GeraldSrivastava, SaurabhHortigüela, María J.Stachewicz, UrszulaHorvai, GeorgeStadie, Nicholas P.Horváth, OttóStadnichenko, AndreyHorvath, RobertStafeev, SergeyHoskins, ClareStagi, LuigiHosseinpour, SamanStalikas, ConstantineHotovy, IvanStamate, EugenHou, BoStamatis, HaralambosHou, Shuhn-ShyurngStan, GheorgheHou, XianghuiStanca, Sarmiza ElenaHousecroft, CatherineStanchina, WilliamHouska, JiriStanciu, Stefan G.Howell, BobStanculescu, AncaHowes, PhilipStańczyk, Wlodzimierz A.Howlader, Matiar R.Starostenkov, MikhailHoyos, MarioStaruch, MargoHristozov, DanailStaub De Melo, Joao VictorHrnjak-Murgić, ZlataStavber, StojanHsiang, Hsing-ISteeneken, PeterHsiao, Chung-DerStefan, MariusHsiao, Hui-HsinStefan, RazvanHsiao, I-LunStefanou, GeorgeHsiao, Pai-YiStehr, Jan EricHsiao, VincentSteinhauer, StephanHsiao, Yu-JenStellato, FrancescoHsu, Jin-CherngStelmachowski, MarekHsu, Yu-KueiStelmachowski, PawełHsueh, Yi-HuangStenbeck, GudrunHu, AnmingStenzel, OlafHu, Ching-YaoStępnik, MaciejHu, HelenStępniowski, WojciechHu, Shang-HsiuStergar, JanjaHu, Teh-MinSthal, FabriceHu, YongStinaff, Eric A.Huang, Cheng-LiangStine, Keith J.Huang, Chia-HuaStirner, ThomasHuang, Chih-ChiaStobiecka, MagdalenaHuang, Chih-ChingStock, UlrichHuang, Chih-FengStodolak, EwaHuang, Chi-HsienStoikov, Ivan Ivanovichhuang, Chin-Pao (C.P.)Stoilova, OlyaHuang, Genin GaryStojek, ZbigniewHuang, Hung JiStolarczyk, AgnieszkaHuang, HungJiStoller, MarcoHuang, Michael H.Stolov, Andrei A.Huang, QijinStolyarov, Vasily S.Huang, RuomengStöwe, KlausHuang, WeixinStrambini, LucanosHuang, XiaodanStrankowski, MichalHuang, Yu-HsiStráský, JosefHubbe, MartinStratakis, EmmanuelHubbe, Martin A.Stratakis, ManolisHuber, TimStraumal, Boris BHubička, ZdeněkStreator, JeffreyHuerta-Angeles, GloriaStrečka, JozefHueso, Jose L.Strehmel, VeronikaHughes, Christopher V.Strein, TimothyHughes, StevenStrelcov, EvgheniHuh, Do SungStrelniker, YakovHuh, Yun SukStride, John ArronHuignard, Jean-PierreStringaro, AnnaritaHumbert, BernardStrini, AlbertoHumbert, ChristopheStrojny-Cieslak, BarbaraHumelnicu, DoinaStrout, Douglas L.Hung, Tai-FengStrunskus, ThomasHung, Yung-TseStuart, Marc C. A.Huntosova, VeronikaStuhr, UweHuot, JacquesStylianakis, MinasHuple, DeepakStylianakis, Minas M.Húska, DaliborSu, Ruey-ChyiHussain, ManwarSu, Wu-ChouHussainova, IrinaSubbaraman, HarishHutchinson, RobinSuber, LorenzaHuynen, IsabelleSubramanian, PalaniappanHwang, InchanSuchaneck, GunnarHwang, Jun YoungSuemune, IkuoHwang, Jun-DarSugai, YuichiHwang, Sung-JooSugawa, KosukeHwang, WanSikSugawara-Narutaki, AyaeHwang, Yoon-HwaeSugiura, YukiHwang, YuhoonSuh, Jun MinHyun, Dong ChoonSujecki, SlawomirIaccino, EnricoSuk, Ji WonIanieri, AdrianaSullivan, Kyle ThomasIannazzo, DanielaSumboja, AfriyantiIatsunskyi, IgorSumby, ChristopherIbáñez, MariaSun, An-ChengIbrahim, ToniSun, Chia-LiangIchikawa, MasakazuSun, DunmeiIchikawa, TakahiroSun, HanjunIchikawa, YoSun, KaiIconaru, Simona LilianaSun, Kien-WenIdris, AdiSun, MengIentile, RiccardoSun, Shih-JyeIervolino, GiuseppinaSun, TaoIglesias, EmiliaSunada, YusukeIglesias, OscarSundaram, SureshIglesias-Martínez, EmiliaSundarrajan, SubramanianIgnaszak, AnnaSuñé, JordiIgnat, MariaSurblé, SuzyIhara, HirotakaSurmeneva, MariaIhlemann, JürgenSuryanto, BryanIjima, HiroyukiSutton, Bruce G.Ikegami, MasashiSuzuki, HiroakiIkemoto, YukaSuzuki, NorihiroIlahi, BouraouiSuzuki, YoshiakiIlan, ShalishSuzuki, YoshioIlanchezhiyan, PugazhendiSverdlov, ViktorIlaš, JanezSvirshchevskaya, Elena V.Iliev, BoyanSvoboda, PetrIllés, ErzsébetSwain, Bhabani SankarIlnicka, AnnaSwaminathan, VenkataramanIm, HyungsoonSwiatkowski, AndrzejIm, JisunŚwierczek, JanImprota, GiovanniŚwietlik, RomanInada, RyojiSyrrokostas, GeorgeInagaki, NorihiroSysoev, VictorInam, FawadSyverud, KristinInfantes-Molina, AntoniaSzabó, GyörgyInkielewicz-Stepniak, IwonaSzabó, TamásInoue, ShuheiSzafraniak-Wiza, IzabelaIoan Voicu, StefanSzala, MateuszIoannis, KandarakisSzaller, ZsuzsannaIoele, GiuseppinaSzałowski, KarolIon, Rodica-MarianaSzczepanowicz, KrzysztofIonescu, EmanuelSzczes, AlexandraIonescu, RaduSzczupak, BoguslawIonita, GabrielaSzczurek, AndrzejIonita, PetreSzekeres, Anna MariaIqbal, ZafarSzepvolgyi, JanosIrato, PaolaSzerb, ElisabetaIrie, MasaoSzewczyk, RomanIrina, FierascuSzilágyi, Imre MiklósIrrera, AlessiaSzlachetko, JakubIsac, LuminitaSzperlich, PiotrIsakov, DmitrySzubert, KarolIshida, TamaoSzybowicz, MiroslawIshige, RyoheiSzyc, ŁukaszIshihara, Keiichi N.Szyja, Bartłomiej M.Ishikawa, RyoSzymanska-Chargot, MonikaIshikawa, YoshieSzymborski, TomaszIslam, Md RashadulSzymczyk, AnnaIsland, JoshuaSzyszkowicz, MieczysławIsland, Joshua O.Tabaran, FlaviuIsola, GaetanoTaboada Antelo, PabloItaka, KenjiTaboryski, RafaelItaliani, PaolaTaboryski, Rafael J.Ito, TakeruTada, RuiIto, TakeshiTadanaga, KiyoharuIturri, JagobaTadepalli, SirimuvvaIvan’kova, Elena M.Tadyszak, KrzysztofIvanets, AndreiTaffa, Dereje HailuIvanishchev, Aleksandr V.Taflove, AllenIvanov, Ilia N.Tagliatesta, PietroIvanov, Vladimir K.Taglietti, AngeloIvanova, TatyanaTaglietti, Angelo MariaIvask, AngelaTagmatarchis, NikosIwan, AgnieszkaTaguchi, YoshihiroIzadyar, AnahitaTaheri, ShimaIžák, TiborTahershamsi, LeiliJablonska, EwaTahir, Muhammad N.Jacak, JanuszTai, Dar-FuJacak, JaroslawTakagaki, AtsushiJacak, LucjanTakano, ToshiyukiJackson, MarkTakayama, OsamuJacquemin, JohanTakazawa, KenJadczak, Joanna N.Takegahara, NorikoJadwicienczak, WojciechTakemori, HiroshiJaffrezic-Renault, NicoleTakeshita, SatoruJakiela, SlawomirTakeuchi, TomonariJakob, GerhardTakshi, ArashJakubec, PetrTalapaneni, SiddulunaiduJakubik, Wiesław P.Talneau, AnneJakubowski, WitoldȚălu, ȘtefanJampaiah, DeshettiTamagnone, MicheleJampilek, JosefTamba, Bogdan IonelJamting, AsaTamulevicius, SigitasJanas, DawidTamura, RuiJanda, PavelTan, JoanneJänes, AlarTan, Swee ChingJang, Ho WonTan, Wai KianJang, HongjeTanabe, TadaoJang, JaewonTanaka, KeijiJang, Jum SukTanaka, TomonariJankauskaite, VirginijaTanemura, KiyoshiJankovsky, OndrejTang, WilkinJankowska, KatarzynaTani-Ishii, NobuyukiJanowska, IzabelaTanini, DamianoJanus, MagdalenaTański, TomaszJanuszko, AdamTappertzhofen, StefanJašek, OndřejTaratula, OlenaJasim, Ali A.Tardy, BlaiseJastrzębska, Agnieszka MariaTariq, MohammadJaszcz, KatarzynaTarrés, QuimJauffres, DavidTartey, SarangJauregui, CatherineTashima, DaisukeJayatissa, Ahalapitiya H.Tasis, DimitriosJechow, AndreasTateishi-Karimata, HisaeJędrysiak, JarosławTatullo, MarcoJedrzejewska-Szczerska, MałgorzataTauböck, TobiasJee, Jun-PilTavakoli, JavadJeerapan, ItthiponTavangarian, FariborzJeleń, PiotrTavares, AnthonyJelliss, PaulTavares, Carlos Jose MacedoJen, Yi-MingTay, Chor YongJena, Himanshu SekharTayabali, AzamJena, Rajeeb K.Taylor, MatthewJenks, WilliamTchekalarova, JanaJensen, Brian D.Tchoukov, PlamenJensen, Gerard M.Tebyetekerwa, MikeJeon, IlTeghil, RobertoJeon, Seog-JinTeixeira, Paulo I.C.Jeong, Chang KyuTeixidor, FrancescJeong, Heon-HoTéllez, CarlosJeong, Jun-hoTemst, KristiaanJeong, Keun-YeongTen Hagen, Timo L.M.Jeong, Sang MoonTennstedt, PierreJeong, Seong-YongTeodoriu, CatalinJeschke, UdoTeodosiu, CarmenJeyadevan, BalachandranTeramoto, NaozumiJi, Ho-IlTerao, KenJiang, DaweiTerasaki, IchiroJiang, JunkeTercjak, AgnieszkaJiang, QiuTereshina-Chitrova, E.A.Jiang, XuchuanTermentzidis, KonstantinosJicsinszky, LászlóTernon, CélineJimenez Migallón, AlfonsoTerranova, Maria LetiziaJimenez-Villacorta, FelixTertiş, MihaelaJin, Hyo-EonTeyssier, JeremieJin, KyoungsukThakur, Basant KumarJing, QingshenThakur, Vijay KumarJing, WenwenThapa, Raj KumarJiu, JintingThavasi, VelmuruganJmerik, ValentinThomas, Morgan L.Jnawali, GirirajThomas, Neil R.Jo, ChangshinThomas, ThresiaJobic, StephaneThompson, Richard L.Joe, DanielThomson, Neil H.Johánek, ViktorThongprayoon, CharatJohansson, ChristerThrall, Brian D.Johnson, Matthew StanleyTian, FurongJohnsson, MatsTian, ZhitingJohnston, Linda J.Tielens, FrederikJolly, PawanTiemann, MichaelJones, JacobTien, Chuen-LinJones, LatheTien, Li-ChiaJönsson-Niedziolka, MartinTietze, RainerJoo, Ji BongTiggelaar, Roald M.Joo, JinmyoungTikhonov, VladimirJood, PriyankaTimko, MilanJoos, JonasTimoshenko, Alexander V.Jordán, ManuelTino, AngelaJordao, LuisaTinti, GemmaJoshi, NiravTiseanu, CarmenJoubert, OlivierTittonen, IlkkaJouini, NoureddineTkac, JanJournet, CatherineTkáč, JánJovanovski, VaskoTkach, OleksandrJózefczak, ArkadiuszTobias, GerardJuarez, PatriciaToikka, AlexanderJudek, JarosławTojo, ConchaJue, Melinda L.Tökesi, KarolyJugessur, AjuTokunaga, EijiJulian-Lopez, BeatrizTokura, YasuhiroJulien, Christian M.Tolstoguzov, AlexanderJulien, FrançoisTomalia, DonaldJulio, Bastos ArrietaTomanek, BoguslawJun, YongseokTomasiunas, RolandasJun, Young-SiTomašovičová, NatáliaJung, DaewoongTomer, Vijay K.Jung, Ho-SupTomić, SimonidaJung, JaehanTomljenovic-Hanic, SnjezanaJung, Min-CherlTomm, JensJung, Yong ChaeTommasi, ImmacolataJung, YongwonTomšič, BrigitaJuodkazis, SauliusToncheva, AntoniyaJurado Sánchez, BeatrizTong, WenmingJurca, TitelTonti, DinoJursich, GregoryTopala, IonutKabanos, ThemistoklisTopoglidis, EmmanuelKabelác, MartinTopuz, FuatKaczmarek, Anna M.Torabi, MohsenKaczmarek, BeataTorgersen, Maria LyngaasKaczmarek, MichałTorigoe, KanjiroKaczorek, EwaTorii, ShuichiKadam, AvinashTorisawa, YusukeKador, LotharToro, Roberta G.Kafarski, PawelToroker, Maytal CasparyKahlert, HeikeToropova, AllaKahng, Yung HoTorre, Maria LuisaKahouli, AbdelkaderTorrens, FranciscoKajitani, TakashiTorres Vaamonde, José EnriqueKakati, NitulTorroba, TomasKakkar, AshokTortello, MauroKako, TetsuyaTortora, PaoloKalantari, KatayoonTortschanoff, AndreasKalantar-Zadeh, KouroshTosa, Monica IoanaKalas, WojciechTosheva, LubomiraKalbáčová, Marie HubálekTosoni, SergioKalinin, VladimirTost, Ramón MorenoKalna, KarolTotaro, GraziaKalska-Szostko, BeataTóth, Ildikó Y.Kamada, KaiTotu, EugeniaKamalakar, M. VenkataTouchy, Abeda S.Kamali, SaeedToudert, JohannKamanina, NataliaTownley, HelenKamenetska, MariaTrammell, Scott A.Kamiguchi, SatoshiTrashin, StanislavKamińska, AgnieszkaTrassin, MorganKaminski, KamilTrens, PhilippeKämmerer, Peer W.Tribello, GarethKamnev, AlexanderTricoli, AntonioKamolz, Lars-PeterTrindle, CarlKampf, NirTrinh, ThuatKan, Chi-waiTripathi, Kumud MalikaKanapitsas, AthanasiosTrivedi, RahulKandammathe Valiyaveedu, SreekanthTrocino, StefanoKaner, RichardTrofimov, EvgenyKaneti, Yusuf ValentinoTronci, GiuseppeKang, HyoTrouillon, RaphaëlKang, KyungtaeTrubiani, OrianaKang, Sung GuTrujillo, CristinaKang, TaejoonTrukhanov, Alex V.Kang, WonkuTruong, Vi-KhanhKanik, MehmetTrusso Sfrazzetto, GiuseppeKano, ShinyaTrusso, SebastianoKantartzis, Nikolaos V.Tryba, BeataKao, Tsung ShengTrybala, AnnaKapcia, Konrad JerzyTrybuła, ZbigniewKapłonek, WojciechTryk, DonaldKarabagias, Ioannis K.Trzaska, JacekKarahan, H. EnisTrzeciak, AnnaKarakasidis, TheodorosTsai, Chen-YenKarakoti, AjayTsai, Ming-JunKaramanis, PanaghiotisTsai, Shiao-WenKarapetis, StefanosTsakova-Stancheva, VesselaKarasawa, SatoruTsalikis, DimitriosKaravasili, ChristinaTsangouri, EleniKarczewski, GrzegorzTsao, Hoi NokKareiva, AivarasTsay, CHien-YieKargl, RupertTschauner, OliverKariuki, BensonTse, William K.F.Karki, Khadga JungTselev, AlexanderKarle, TimothyTseng, Wei-LungKároly, LázárTseng, Zong-LiangKarousou, EugeniaTserepi, AngelikiKarpov, SergeyTsigaridas, GeorgiosKarpov, VictorTsilipakos, OdysseasKarthäuser, SilviaTsitsilianis, ConstantinosKarthik, RajTsitsilonis, Ourania E.Karwowska, EwaTsivintzelis, IoannisKaseem, MosabTsoukalas, DimitrisKashaninejad, NavidTsui, TingKashimura, KeiichiroTsuji, YasuhideKashiwagi, ShinichiroTsujimoto, YoshihiroKasiotis, KostasTsukamoto, ShiroKasnatscheew, JohannesTsurimaki, YoichiroKassner, MichaelTsurkan, Mikhail V.Kaszkur, ZbigniewTsuru, ToshinoriKataria, SatenderTsutsui, MakusuKaterski, AtanasTsutsumi, NaotoKatin, KonstantinTsutsumi, OsamuKato, KatsuyaTsvetkov, NikolaiKato, Takamitsu A.Tu, MinKato, TakeshiTubaro, CristinaKatoh, KeiichiTuci, GiuliaKatoh, RyuziTuma, DirkKatz, EvgenyTunesi, MartaKauppinen, Esko I.Tung, Chun-WeiKaushik, Nagendra KumarTung, Shih-HuangKaushik, NehaTung, Tran ThanhKavousanakis, MihalisTurco, AntonioKawakami, HiroshiTurco, RosaKawasaki, HideyaTürk, MichaelKazda, TomášTurkmen, MustafaKazek-Kęsik, AlicjaTurkowski, Volodymyr M.Kaznelson, BorisTurner, Raymond J.Kazuhito, SatomuraTuros, EdwardKelarakis, AntoniosTurren-Cruz, Silver-HamillKelley, DavidTuti, SimonettaKelley, StevenTwardowski, AndrzejKelly, HelenaTyan, Yu-ChangKenanakis, GeorgeTyczkowski, JacekKennedy, David C.Tyliszczak, BożenaKenry, K.Tylkowski, BartoszKepinska, MartaTyszkiewicz, CumaKeplinger, TobiasTzanavaras, Paraskevas D.Keppert, MartinTzeng, YonhuaKerman, KaganTzetzis, DimitriosKern, FrankTzounis, LazarosKetoja, JukkaUccelletti, DanielaKhadka, Dhruba B.Uchida, SatoshiKhalakhan, IvanUeda, TaroKhalid, AtaUeno, KoseiKhalilov, UmedjonUetani, KojiroKhalilzadeh, Mohammad A.Ujihara, MasakiKhan, FasihullahUl Haq, RizwanKhan, Md. ShakhaoathUlanski, PiotrKhan, Omar F.Uldrijan, StjepanKhan, ZiauddingUlewicz, MałgorzataKharlamova, Marianna V.Ulloa, Sergio E.Khasanov, RustemUm, Doo-seungKhenner, MikhailUm, Han-DonKhisamutdinov, EmilUmek, PolonaKholodnyak, DmitryUmerska, AnitaKhonina, SvetlanaUnnithan, Ranjith RajasekharanKhoshmanesh, KhashayarUnno, MasafumiKiani, AmirkianooshUnterreiner, Andreas-NeilKiciński, WojciechUnuma, HideroKidalov, SergeyUrbanczyk-Lipkowska, ZofiaKiesewetter, Dale O.Urbano, Ana MargaridaKim, Ae RhanUrbassek, HerbertKim, BongjuUrbieta, AnaKim, Byeong-SuUrsini, Cinzia LuciaKim, Byoung SuhkUshakova, Elena V.Kim, Chang-HyunUskoković, VukKim, Cheol GiUsov, NikolaiKim, Choong-unUspal, WilliamKim, ChoonsooUsui, HidetomoKim, DaehwanUsui, HiroyukiKim, DaewonUtsumi, ShigenoriKim, Dai-SikUznanski, PawelKim, Do YoungUzunalli, GozdeKim, Dong WukV. Makarov, SergeyKim, DonghyunVaculovičová, MarketaKim, Dong-JooVaerenbergh, Stefan VanKim, Eun KyuVafaei, SaeidKim, FelixVafapour, ZohrehKim, Hae-WonVahabi, HenriKim, Han-JungVaiano, VincenzoKim, Hwan D.Vaikuntanathan, VisakhKim, Hyug-HanVajtai, RobertKim, Hyun ChanVakros, JohnKim, Hyun JaeValais, IoannisKim, Hyun SooValant, MatjazKim, Hyun TakVale, NunoKim, HyungsikValente, Artur J.M.Kim, HyungsupValentini, FedericaKim, HyunseokValiyaveettil, SureshKim, Hyun-SikVallée, ChristopheKim, HyunsungValsami-Jones, EugeniaKim, Il TaeVan Bennekom, Wouter P.Kim, Jae-HwanVan Den Bergh, HubertKim, JaeyounVan Der Weijden, RenataKim, JeesuVan Herk, AlexKim, JehyungVan Schepdael, AnnKim, JeongyongVancaeyzeele, CédricKim, JihoonVandebriel, RobKim, JincheolVanetsev, AlexanderKim, JinsooVankayala, RavirajKim, Jong HyunVanko, GabrielKim, Joo HyunVáradi, CsabaKim, Jung J.Varadwaj, Pradeep R.Kim, Jung RaeVarga, TamasKim, Keun-SooVarga, ZoltanKim, Ki KangVarganici, Cristian-DragosKim, Ki SuVarikuti, SanjayKim, KikangVarma, Rajender S.Kim, Kwang SVarna, MarianaKim, Kyung HoVasanelli, AngelaKim, KyungBaeVashist, ArtiKim, Kyung-MinVashurin, ArturKim, LauraVasilakaki, MariannaKim, MinVasile, Bogdan StefanKim, Min-CheolVasiljevic, NatasaKim, Min-SooVasilopoulou, MariaKim, MinsuVasilyev, AlexanderKim, Myung-GilVattikuti, Surya Veerendra PrabhakarKim, Nam HoonVázquez De Aldana, Javier RodríguezKim, Ok-SuVecsernyés, MiklósKim, SangVedarajan, RamanKim, SanghoonVédrine, Jacques C.Kim, Sang-JaeVejpravová, Jana KalbacovaKim, SejeongVélaz, ItziarKim, Seok KiVelazquez-Perez, Jesus EKim, SeonahVelena, AstridaKim, Seong-GonVelha, PhilippeKim, SoaramVelkov, TonyKim, SooVelotta, RaffaeleKim, Soo YoungVenditti, IoleKim, SooyeonVenkatesan, JayachandranKim, SoyounVentura, Cinzia AnnaKim, Sung Y.Vercelli, BarbaraKim, SungjunVerdejo, BegoñaKim, Sung-KonVerdú-Andrés, JorgeKim, Tae HeonVeres, MiklosKim, TaehoonVergara, AlessandroKim, Tae-hwanVergaro, VivianaKim, Tae-HyungVergaz, RicardoKim, YounghoonVerheijen, Marcel AKim, Young-JinVerma, Navin KumarKim, Young-RokVerma, PragyaKim, Yu KwonVerma, SannyKimura, MutsumiVernardou, DimitraKimura, YoshiharuVertruyen, BenedicteKioseoglou, GeorgeVertuccio, LuigiKireev, DmitryVetyukov, YuryKirm, MarcoVia, BrianKiryukhantsev-Korneev, Ph.V.Vicente, Miguel A.Kiselev, AntonVicenzo, AntonelloKishimoto, SatoshiVidal, HilarioKiss, JánosVidic, JasminaKitagawa, JiroVidu, RuxandraKitahama, YasutakaVikraman, DhanasekaranKitamura, MasatoshiVilà, AnnaKitamura, TakayukiVila, CarlosKitaura, RyoVilanova, XavierKitazawa, TakafumiVilardi, GiorgioKityk, Andriy V.Vilela, CarlaKitzerow, Heinz Siegfried R.Villa, IreneKlang, VictoriaVillacampa Naverac, María BelénKlein, AxelVillaluenga, IruneKlein, Lisa C.Villar, MarceloKlein, SusanneVillares, AnaKlemens, KelmVillaverde, Juan JoséKlimin, SergeiVinardell, MariaKling, RainerVinattieri, AnnaKlini, ArgyroVincent-Bonnieu, SebastienKlinkova, AnnaViñes, FrancescKliros, George S.Vingolo, Enzo MariaKloskowski, TomaszVinnik, DenisKo, ChanghyunVinogradov, Alexey P.Ko, Seung HwanVinogradov, VladimirKo, SeunghwanVirgili, TersillaKobayashi, RikaViricelle, Jean PaulKobayashi, TaishiVirkki, JohannaKobayashi, TatsuyaVisai, LiviaKobayashi, YoshiroVisaveliya, NikunjkumarKobayashi, YuichiroVisco, AnnamariaKoblischka, Michael R.Viskadourakis, ZachariasKoblischka-Veneva, AnjelaVismara, ElenaKochanowicz, MarcinViter, RomanKochev, NikolayVitkova, VictoriaKocik, MarekVitrik, OlegKoduru, JanardhanVittadello, MicheleKoetz, JoachimVittadini, AndreaKohl, YvonneVittori Antisari, MarcoKohler, MichaelVittoria, AntonioKohri, MichinariVivas Mejia, PabloKoinkar, PankajViviani, MassimoKoizumi, HiroyasuVivo, PaolaKojima, ChieVizureanu, PetricǎKojima, SeijiVlachos, DimitriosKojima, ToshikatsuVlachou, MarilenaKojitani, HiroshiVleugels, JozefKokenyesi, SandorVochita, GabrielaKokkoris, GeorgeVochozka, MarekKokol, VanjaVogiatzis, GeorgiosKoksharova, Olga A.Vogt, MartinKolaric, BrankoVohra, VarunKolasinski, Kurt W.Voicu, Stefan IoanKolesnikov, Ilya E.Voirin, GuyKöller, ManfredVokoun, DavidKołodyńska, DorotaVoliani, ValerioKolosnjaj-Tabi, JelenaVOLK, JanosKołtunowicz, Tomasz NorbertVolker, KörstgensKomaguchi, KenjiVolkov, YuriKondo, TakahiroVolodymyr, TkachenkoKong, JaeminVolonterio, AlessandroKong, Kien VoonVolovlikova, OlgaKong, LingxueVon Bardeleben, Hans J.Kononenko, Oleg V.Voronenkov, VladislavKónya, ZoltánVostrikova, Kira E.Koo, JahyunVoz, CristobalKoonce, MichaelVračko, MarjanKoos, Antal A.Vrcek, Ivana VinkovicKopeček, JaromírVuong, Thu V.Kopecký, VladimírWada, TohruKopel, PavelWadati, HirokiKordatos, KonstantinosWagner, PawelKorn, TobiasWajima, TakaakiKorolkov, Ilya V.Walach, WojciechKorolovych, VolodymyrWałajtys-Rode, ElżbietaKőrösi, LászlóWalker, GregKorotcenkov, GhenadiiWalker, JulianKorsunsky, Alexander M.Walper, ScottKorzeniewska, EwaWalsh, Laurence J.Korznikova, ElenaWalther, ThomasKos, ŠimonWan, YatingKosa, GaborWan, YuanKoshimizu, MasanoriWang, BinKoskinen, PekkaWang, Chiu-YenKoslowski, ThorstenWang, CuicuiKosmulski, MarekWang, DanlingKostopoulou, AthanasiaWang, DepengKotek, JanWang, Di-YanKotsiomiti, EleniWang, Dong HwanKotsuchibashi, YoheiWang, FengKoumoulos, Elias P.Wang, GuankuiKounatidis, IliasWang, HaiyangKourkoumelis, NikolaosWang, HanKousal, JaroslavWang, Hsiang-ChenKoutavarapu, RavindranadhWang, Jian-WenKoutselas, IoannisWang, JieKoutsogeorgis, DemosthenesWang, JunKoutsokeras, LoukasWang, KunleiKoutsoukos, PetrosWang, Li-ShengKoutsouris, Dimitris DionissiosWang, MingsongKoutzarova, TatyanaWang, Paul C.Kováč, JaroslavWang, Peng-YuanKovacheva, DanielaWang, ShapengKovacs, GaborWang, Shau-ChunKovalchenko, Andrii M.Wang, WenxinKovalevsky, Andrei V.Wang, WenzhangKowalak, StanisławWang, XiaoweiKowalczyk, AgataWang, XizuKowalczyk, PawełWang, Yan-HsiungKowalik, PawełWang, YanxingKowalska, Aneta AnielaWang, Ying-JanKowalska, EwaWang, YuKoyama, YasuhitoWang, YueshengKozak, MartinWang, Yu-QingKozempel, JánWang, ZhiguangKozlova, EkaterinaWang, ZhiliangKozlovskiy, ArtemWang, ZhuangKozyreff, GregoryWarcholiński, BogdanKozyukhin, Sergey A.Wardhono, Endarto Y.Kralisch, DanaWark, MichaelKralj, SamoWarmuzek, MałgorzataKralj, SlavkoWarwick, Michael E.A.Kramberger, ChristianWasiak, Michał L.Krasnoslobodtsev, Alexey V.Wasilewski, TomaszKratochvilova, IrenaWasisto, Hutomo S.Kratzer, MarkusWasisto, Hutomo SuryoKrause, Hans-JoachimWatanabe, FumiyaKrawczyk, PiotrWatanabe, MotonoriKrawiec, MariuszWatarai, HitoshiKreouzis, TheoWaters, MichaelKrezhov, KirilWatkins, Anthony NealKristl, MatjažWatzky, MurielleKrólczyk, GrzegorzWawrzkiewicz, MonikaKrólicka, AgnieszkaWeaver, John B.Królicka, AleksandraWebb, Lauren M.Kroll, PeterWeglarz, WladyslawKrompiec, StanisławWei, Hsien-HungKrowne, C. M.Wei, KongchangKrstulović, NikšaWei, Qi-HuoKrumeich, FrankWei, ZongsuKrupa, Kristen A.Weidlich, Ernst-RudolfKrymsky, Valeriy V.Weimann, Nils G.Krysa, AndreyWeinberger, PeterKrzanowski, JamesWeiss, Clemens K.Krzanowski, James E.Wejrzanowski, TomaszKrztoń-Maziopa, AnnaWen, JianmingKu, Pei-ChengWen-Jeng, HoKubát, PavelWenzel, DanielaKubli, MartinWertz, Philip W.Kubo, TakanoriWestover, Andrew S.Kuchler-Bopp, SabineWhite, AlvinKüçüköz, BetülWhitehead, Debra E.Kuddannaya, ShreyasWhittington, StuartKudelski, AndrzejWia̧cek, Agnieszka EwaKudłak, BłażejWIbowo, Rachmat AdhiKudr, JiriWie, Jeong JaeKudryashov, Sergey I.Wieczorek, Władysław G.Kudrynskyi, ZakharWiemann, MartinKühnel, DanaWiemer, ClaudiaKujawa, JoannaWierer, Jonathan J.Kujawski, WojciechWieszczycka, KarolinaKukli, KaupoWiglusz, Rafał JakubKukułka, WojciechWijnholds, JanKulagin, RomanWijnhoven, SusanKulbacka, JulitaWilkins, Richard T.Kulnitskiy, Boris A.Williams, David S.Kumar Thakur, VijayWilliams, TravisKumar, AnilWilson, Andrew J.Kumar, BijandraWilson, Justin J.Kumar, HarishWilts, BodoKumar, RameshWinter, DominicKumar, SandeepWiraja, ChristianKumar, SanjeevWiśniewski, MarekKumeria, TusharWitowski, AndrzejKunc, JanWittemann, AlexanderKunčická, LenkaWittenberg, NathanKundu, TanayWittmann, JürgenKung, Chung-WeiWłodarczyk, PatrykKunitake, MasashiWoeltje, MichaelKunli, GohWojciechowski, SzymonKuo, Chi-ChingWojcieszak, RobertKuo, Chien-NanWojnar, PiotrKuo, ShiaoweiWojnarowicz, JacekKuo, Tsung-RongWojtkowska, MałgorzataKupfer, StephanWolf, KatarinaKurajica, StanislavWołowiec, StanisławKureha, TakumaWong, BryanKurihara, SeijiWong, Clarence T. T.Kuřitka, IvoWong, Danny K.Y.Kurlyandskaya, Galina V.Wong, GarryKurniawan, Nicholas A.Wong, HarrisKuroda, TakashiWong, Ka-LeungKuroiwa, KeitaWong, Pamela T.Kurokawa, HidekiWood, Troy D.Kurzydłowski, Krzysztof J.Woodward, Robert T.Kushibiki, ToshihiroWoon, Wei-YenKusior, AnnaWright, CatherineKustov, LeonidWróbel, JanKusukawa, TakahiroWrobel, RafałKuttner, ChristianWu, BingKutyła, DawidWu, Chia-WenKuzhir, PavelWu, Chin-SanKuzhir, Polina P.Wu, Gwo-MeiKuzma, LukaszWu, HongjingKuzmik, JanWu, Hwai-ChungKuzmin, AlexeiWu, Jerry J.Kuznetsov, DenisWu, Jiang-BinKuznetsova, Iren E.Wu, Jing-YunKuzuya, AkinoriWu, Ming-ChungKvitek, LiborWu, Peter K.Kwan, Hiu-yeeWu, ShengyunKweon, Oh-KyeongWu, Shin-TsonKwiatkowski, MirosławWu, Xin-PingKwon, Hyuck-InWunderlich, WilfriedKwon, Hyuk-JunWurster, StefanKwon, Il-KeunWuu, Dong-SingKwon, Kwang-HoWyman, IanKwon, Nam HeeWysokinski, Karol IzydorKwon, Soon-HongWysokowski, MarcinKwon, WoosungXenakis, AristotelisKwon, Young-WanXi, KaiKyzas, GeorgeXia, TianKyzioł, AgnieszkaXia, WeiLa Ferla, BarbaraXiao, ShuminLa Flor, Silvia DeXiao, XinxinLa Parola, ValeriaXie, FengweiLa Spina, RitaXing, GengmeiLa, Duc DuongXiong, HaoLaaksonen, PäiviXiong, RuiLaarmann, TimXu, GuLabat, BeatriceXu, JinzeLabate, LucaXu, LichongLachowicz, DorotaXu, TaoLaganà, AldoXu, TianhuaLagoa, RicardoXu, WenwuLagunas Tergarona, AnnaXu, XiaoxueLahiri, AbhishekXu, YongLahtinen, JoukoXuan, ShouhuLai, Chih-HsienYablonsky, Gregory S.Lai, Jui-YangYadav, HemrajLai, MinliangYadav, Hemraj MLai, Yi ShengYadav, Raghvendra SinghLaidani, Nadhira BensaadaYadav, SushmaLaïk, BarbaraYagi, HiroshiLakshminarayana, GandhamYahiaoui, RiadLam, EdmondYakhvarov, DmitryLam, Kwok-hoYakimova, LuidmilaŁamacz, AgataYakimova, RositsaLambin, PhilippeYakunin, AlexanderLambrecht, WalterYamada, TakeoLamouroux, EmmanuelYamaguchi, AkinobuLamponi, StefaniaYamaguchi, MakotoLampre, IsabelleYamaguchi, MasahikoLan, ShoufengYamaguchi, MasatakeLan, Wen-HowYamaki, KazuhiroLan, YuchengYamamoto, GoLanceros-mendez, SenentxuYamamoto, Hiroshi M.Lančok, JánYamamoto, YusukeLanger, Jerzy J.Yamato, TakehikoLang-Olip, IngridYamauchi, MitsuakiLanterna, AnabelYamauchi, YusukeLappan, UweYan, DayunLaptev, AlexanderYanagida, SayakaLaranjo, MafaldaYáñez-Vilar, SusanaLarrañaga, AitorYang, Cheng-FuLartigue, LenaicYang, Chen-ILashbrook, MarkYang, Chia-MinLassalle, Henri-PierreYang, GeLászló, KrisztinaYang, HongshunLattanzi, WandaYang, HongtaLatterini, LoredanaYang, HsiharngLattuada, MarcoYang, Hung-WeiLaura, Romero-ZeronYang, In-SangLaurenti, MarcoYang, LilyLauritano, DorinaYang, MinyangLavrentovich, Oleg D.Yang, Seung-JaeLaw, Cheryl SuwenYang, Ta-ILayek, BuddhadevYang, ZongyinLázár, IstvánYANGUI, AymenLazarus, NathanYannopoulos, SpyrosLazzara, GiuseppeYano, HajimeLazzari, MassimoYano, MitsuakiLazzeri, MicheleYao, Da-JengLe Breton, Jean-ChristopheYao, JieLe Gal La Salle, AnnieYaroshchuk, OlegLe Ouay, BenjaminYasa, OncayLe Quynh, HoaYashchenok, AlexeyLe, DuyYates, MatthewLebeau, BénédicteYazdani Nezhad, HamedLebeau, LucYazdi, ImanLebedev, Alexander AlexandrovichYazdimamaghani, MostafaLeBlanc, Roger M.YE, EnyiLebron, José AntonioYehezkeli, OmerLeclère, PhilippeYen, Hung-JuLee, Adam F.Yeo, Min-KyeongLee, AlbertYeong, Wai YeeLee, BongmookYeum, JeongLee, ChangguYilmaz, MehmetLee, Cheng-ChungYim, ChangyongLee, Cheng-IYin, ZhouyangLee, ChiYokochi, AlexandreLee, Chia-HungYokohira, MasanaoLee, Chih-HaoYokose, SatoshiLee, DaehoYokota, ShingoLee, Dai-SooYong, Kar WeyLee, Dong UnYong, XinLee, DonghyunYoo, Doo-YeolLee, DongjuYoo, JeeyoungLee, DongkyuYoon, HyeonseokLee, DoojinYoon, Hyun ChulLee, EunsongyiYoon, Jong-GulLee, Gang HoYoon, JongwonLee, GeunsikYoon, Seog-YoungLee, GibaekYOSHIDA, KojiLee, Gwan-HyoungYoshida, TakashiLee, Hae-HyoungYoshimatsu, KoheiLee, Hae-SeokYoshino, KenjiLee, Han EolYoshino, ToshihikoLee, Hian KeeYou, ShujieLee, Hiang KweeYou, ZhenjiangLee, Hsien-MingYoun, Duck-hyunLee, Hsin-ChenYoung, Sheng-JoueLee, HyoungsoonYu, HakkiLee, IlkeunYu, LuLee, Jae SungYu, MeihuaLee, JaehanYu, QiumingLee, JaehongYu, TaekyungLee, JaehwangYu, WilliamLee, Jae-UngYuan, Chi-TsuLee, Jea UkYuan, Chiun-JyeLee, JeongmiYuan, GuanghuiLee, Jeong-SooYubero, FranciscoLee, Jung MinYufera, AlbertoLee, Jung SeokYuk, Jong MinLee, JungmanYuldashev, ShavkatLee, JunseongYum, Jun HoLee, JunseopYun, HongseokLee, Kew-HoYun, Seok JoonLee, Kyoung G.Yun, Young SooLee, Min WookYusa, Shin-ichiLee, MooyeonYuzhalin, Arseniy E.Lee, Rong-HoZabeida, OlegLee, Sang-HanZabihi, OmidLee, SangkilZablotskii, VitaliiLee, Sang-LaeZairov, RustemLee, Sang-yupZakaria, Mohamed BarakatLee, SejoonZakharenko, Alexander M.Lee, SeunggeolZakharov, Alex V.Lee, SeunghyunZaleska-Medynska, AdrianaLee, Soo ChoolZallo, EugenioLee, SunghoZambon, DanielLee, SunheeZampetti, EmilianoLee, SzetsenZampolli, StefanoLee, TaehunZangari, GiovanniLee, TaekZannotti, MarcoLee, Ting-KuoZapotoczny, SzczepanLee, Tse-MinZaraska, LeszekLee, Woo-KyungZareba, Jan K.Lee, Yoon HoZarrin, HadisLee, Young-ChulZarrintaj, PayamLEGRAND, François-XavierZatsepin, Anatoly F.Lehnherr, DanZavoianu, RodicaLehr, Claus-MichaelZawala, JanLei, WenZazpe, RaulLeineweber, AndreasZdarta, JakubLeipzig, Nic D.Zdenek, SloukaLeitao, Diana C.Zdrojek, MariuszLeitner, JindrichZdunek, KrzysztofLeitner, MichaelZekonyte, JurgitaLeiva, CarlosŻelechowska, KamilaLellouche, Jean-PaulZelkó, RománaLemercier, GillesZelzer, MischaLemos, JuliaZemek, JosefLemus, RanulfoZeng, HuangLenaghan, ScottZeng, ShuwenLengalova, AnezkaZeng, XiangkangLennartz, MichelleZerulla, DominicLenser, ChristianZestos, Alexander G.Leon, LorraineZgórka, GrażynaLeong, David T.Zgură, IrinaLeonski, WiesławZhan, TaoLeporatti, StefanoZhang, Can YangLepore, MariaZhang, ChaoLereu, Aude L.Zhang, DuoLerouge, FredericZhang, EnxiaLesar, BoštjanZhang, JunyingLesiák-Orłowska, B. LesiakZhang, KailiLeskelä, MarkkuZhang, QiangzheLesnyak, VladimirZhang, QichunLesov, IvanZhang, QingzheLessard, BenoitZhang, RunLeszczyńska, AgnieszkaZhang, SenLetek, MichalZhang, WeiLétoublon, AntoineZhang, WujieLettieri, StefanoZhang, XiangLeung, KaHoZhang, YaohongLeuteritz, AndreasZhang, YaoxinLévêque, PatrickZhang, YiLevin, Oleg VladislavovichZhang, YueLevine, MindyZhang, YunyanLevine, Mindy S.Zhang, ZhidongLewandowski, MikołajZhao, FengLewandowski, WiktorZhao, HongLewinski, JanuszZhao, Jiangbo (Tim)Lewis, David A.Zhao, SongruiLhuillier, EmmanuelZhao, XiuboLi Voti, RobertoZhao, YudaLi, GangZhao, YunfengLi, GuoshengZharkov, SergeyLi, JasonZhen, QiLi, JiantongZheng, KaiLi, JianxiongZheng, KunLi, JiashenZheng, XintingLi, JinghuaZheng, YuebingLi, Kuo-TsengZheng, ZhongLi, MingxingZhong, LiLi, XiaoZhou, JiajiaLi, XinmingZhou, JiangbingLi, Yi-ChenZhou, Jian-PingLi, YifanZhou, KunLi, YingZhou, ShengjunLi, YiwenZhou, YiqunLi, ZiyuanZhou, YouLiang, AnZhu, HongyangLiang, Chi-TeZhu, HuiLiang, QijieZhu, JiadengLiang, Yuan-ChangZhu, MinminLiang, YucangZhu, ZhiguangLianos, PanagiotisZhuangqiang, GaoLiao, Jiunn-DerZhuravlev, VictorLiao, Wei SsuZiąbka, MagdalenaLiao, Ying-ChihZiegler-Borowska, MartaLiaparinos, PanagiotisZielasek, VolkmarLider, AndreyZielinska, AleksandraLiebscher, MarcoZielinska, BeataLieder, MarekZielińska-Jurek, AnnaLiedl, TimZieliński, AndrzejLien, Der-HsienZielinski, PiotrLiesiene, JolantaZierold, RobertLignos, IoannisZignani, Sabrina CampagnaLikodimos, VlassiosZille, AndreaLillo-Ródenas, Maria ÁngelesZilm, PeterLim, Dong ChanZimbone, MassimoLim, Eun-KyungZimmer, ThomasLim, JongwooZimmerman, WilliamLim, Ki-TaekZinelis, SpirosLim, Min-CheolZinin, Pavel V.Lim, Sang KyooZiora, Zyta MariaLim, SoomanZiyatdinova, GuzelLima, RuiZmudzki, PawelLima, Rui A.Zobi, FabioLimiti, ErnestoZoltán, PásztiLimongi, TaniaZou, HaiyangLin, Chao-SungZozulya, AlexeyLin, Chia-FengZschech, EhrenfriedLin, Chia-HerZubitur, ManoliLin, Chia-HuaZucali, MicheleLin, Chien-hongZuccari, GuendalinaLin, Chun HongZukowski, WitoldLin, Chun-RongŽumer, SlobodanLin, Der-YuhZuo, JianLin, HaishuŽupanič, AnžeLin, HanZurlo, GiuseppeLin, Hsiu-MeiZytkiewicz, Zbigniew

